# A comparative study of Sumudu HPM and Elzaki HPM for coupled Burgers’ equation

**DOI:** 10.1016/j.heliyon.2023.e15726

**Published:** 2023-05-01

**Authors:** Mamta Kapoor, Varun Joshi

**Affiliations:** a Department of Mathematics, Lovely Professional University, Phagwara, Punjab, 144411, India

**Keywords:** Coupled 1D and 2D Burgers' equations, Sumudu and Elzaki transform, HPM, Error analysis, Convergence analysis

## Abstract

The two hybrid algorithms Sumudu HPM and Elzaki HPM are used in the current study to tackle coupled Burgers' equations and produce accurate results. To demonstrate the validity of the given approaches, three instances are used. Applying Sumudu HPM and Elzaki HPM yields the same approximate and exact answers in all of the examples taken into consideration, which is proved with the help of the accompanying figures. It attests to the entire acceptance and accuracy of the solutions produced by these methods. The proposed regimes also have error and convergence analyses available. The current analytical regimes offer a more effective method of handling partial differential equations than the intricate numerical systems. It is also asserted that exact and approximation solutions are compatible. Also announced is the planned regime's numerical convergence.

## 1 Introduction

**Figure:**
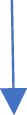

### 1.1 Coupled 1D and 2D Burgers’ equations

The following are the 1D coupled Burgers' equations' governing equations [1,2]..

(1.1) $$\theta_{t}+\delta \;\theta_{xx}+\eta \;\theta \;\theta_{x}+\alpha \;{\left(\theta \eta \right)}_{x}=0\text{,}$$

(1.2) $$\eta_{t}+\mu \;\eta_{xx}+\xi \;\eta \;\eta_{x}+\beta \;{\left(\theta \eta \right)}_{x}=0\text{.}$$

The following is a 2D nonlinear unsteady coupled viscous Burgers' equation [3].:

(1.3) $$\frac{\partial \theta }{\partial t}+\theta \;\frac{\partial \theta }{\partial x}+\eta \;\frac{\partial \theta }{\partial y}=\left(\frac{1}{Re}\right)\left(\frac{\partial^{2}\theta }{\partial x^{2}}+\frac{\partial^{2}\theta }{\partial y^{2}}\right)\text{,}$$

(1.4) $$\frac{\partial \eta }{\partial t}+\theta \;\frac{\partial \eta }{\partial x}+v\;\frac{\partial \eta }{\partial y}=\left(\frac{1}{Re}\right)\left(\frac{\partial^{2}\eta }{\partial x^{2}}+\frac{\partial^{2}\eta }{\partial y^{2}}\right)$$

Equations (1.1)–(1.4) are provided as the coupled Burgers' equations respectively. Esipov [4] developed coupled viscous Burgers' equations to explore model of poly-dispersive sedimentation. Burgers' and Cole pointed out the importance of Burgers' equation for explaining a number of phenomena, such as a mathematical prototype of turbulence and approximation theory of flow brought on by shock wave travelling through a viscous fluid flow. Kaya [5] used decomposition method for this equation and obtained a convergent power series-shaped solution for the homogeneous and nonhomogeneous coupled Burgers' equation. The coupled Burgers' equation was tackled by Khater et al. [6] via Chebyshev Spectral Collocation Method to provide numerical solutions. Via Adomian-Pade method, Dehghan et al. [7] were able to arrive at numerical approximation of coupled Burgers' equation. Via modified extended tanh function approach, Soliman [8] was able to arrive at precise solution to coupled Burgers' problem. VIM was used in Ref. [9] to solve coupled and 1D Burgers' equations. Numerous researchers have attempted to tackle coupled Burgers' equation to fetch smooth approximations due to coupled Burgers' equation's significance in fields of science and engineering; some of these works are shown here. I-LFDM, a concept of logarithmic FD approach, was developed by Srivastava et al. [10] to numerically solve 1D coupled Burgers' equation. To deal with Burgers' type equations numerically, Mittal and Jiwari [11] utilized PDQM approach. Resulting ODEs system was solved via RK $4^{th}$-order method. By utilizing idea of cubic B-spline collocation regime, Mittal and Arora [12] offered numerical solution of coupled 1D viscous Burgers' equation. Fourier pseudo-spectral approach was employed by Rashid and Ismail [13] to solve a 1D coupled Burgers' equations. GDQM was put forth by Mokhtari et al. [14] to tackle coupled Burgers' equation numerically. Novel Lattice Boltzmann model was put forth by Lai and Ma [15] for solving coupled non-linear Burgers' equation. Trigonometric B-spline technology was used by Salih et al. [16] to get a numerical solution to coupled viscous Burgers' problem. To tackle 1D coupled Burgers' equation, Raslan et al. [17] adopted collocation method created by quintic B-spline basis function. To solve coupled Burgers' equation numerically, Liu et al. [18] adopted collocation approach based on Barycentric form of Lagrange interpolation polynomial. A unique LBM was used by Li et al. [19] to solve coupled Burgers' equation. Bhatt and Khaliq [20] used $4^{th}$-order compact techniques to numerically solve coupled Burgers' equation. An implicit FD approach was used by Srivastava et al. [21] to solve coupled Burgers' equation.

Analytical solution to 2D coupled Burgers' equation was introduced by Fletcher [22] via Hopf-Cole transformation. Due to coupled Burgers' equation's significance in numerous scientific and engineering domains, numerous researchers have been able to discover its numerical solution. A sample of their work is shown up front. For solution of 2D coupled Burgers' equation, Tamsir et al. [23] used DQM methodology created by exponential MCB-spline, and they also presented a stability analysis of matrix method. 2D coupled Burgers' equation was solved by Shukla et al. [24] via MCB-DQM, and reduced ODE set was resolved via SSP-RK54 methodology. DTM was used by Abazari and Borhanifar [25] to arrive at a numerical solution to 2D coupled Burgers' problem. To solve 2D coupled viscous Burgers' equation, Mittal and Jiwari [26] applied DQM. A fully implicit FD scheme was developed by Bahadir [27] to solve 2D coupled Burgers' equation. Cubic spline function method was used by Jain and Holla [28] to find solution to coupled Burgers' problem.

Adomian Decomposition Technique was employed by Zhu et al. [29] to arrive at numerical solution of 2D coupled Burgers' problem. To deal with coupled 2D Burgers' equation, Fan et al. [30] developed a novel method that combines local RBF-based Collocation method and FTIM. LDM was used by Majid Khan [31] to tackle coupled 2D Burgers' equation. HPM was used by Hizel and Kucukarslan [32] to deal with coupled (2 + 1)D Burgers' equation. For coupled 2D Burgers' equation, Srivastava et al. [33] employed implicit I-LFDM approach. Coupled Burgers' equation was numerically solved by Srivastava and Tamsir [34] via Crank-Nicolson semi-implicit technique. Zhanlav et al. [35] created a system to tackle coupled 2D Burgers' equation and presented a higher-order explicit FD regime to deal with 2D heat equation, which has a $4^{th}$-order space variable approximation and a $2^{nd}$ order time variable approximation. To tackle coupled 2D Burgers' equation, Liao [36] suggested a $4^{th}$-order compact FD methodology. This approach was based on 2D Hopf-Cole transformation, which converts 2D Burgers' equation system into a linear heat equation. To deal with coupled 2D Burgers' equation, Zhao et al. [37] used LDG FEM. This method was based on 2D Hopf-Cole transformation, which converted 2D Burgers' equation system into a linear heat equation before being solved by LDG FEM. Numerical investigation for an inclined rotating disc was provided by Sheikholeslami et al. [38]. Optimization of a circular-wave cavity under conditions of natural convection heat transfer was described by Hatami et al. [39]. Concept of natural convection heat transfer in a cavity filled with nanofluid was introduced by Tang et al. By utilizing nano fluid and RSM analysis, Hatami and Jing [40] introduced a wave direct absorption solar collector. Hatami et al. [41] first proposed the idea of optimizing a lid-driven T-shaped porous cavity to enhance eat transmission from nano-fluid mixed convection. Concept of designing a micro channel was presented by Zhou et al. [42] and its time-efficiency was examined.

### 1.2 Sumudu transform

Watugala gave notion of Sumudu transform in 1993, which was used to tackle problems of engineering control [43]. Later on, notion of Sumudu transform was extended by Watugala in 2002 from one variable to two variables. Equations (1.5)–(1.10) are provided regarding Sumudu and Elzaki transforms respectively.

Sumudu Transform is defined as follows [43,44]:

(1.5) $$F\left(\theta \right)=S\left[f\left(t\right),\theta \right]=\frac{1}{\theta }\int_{0}^{\infty }e^{-\frac{t}{\theta }}\;f\left(t\right)dt$$

f(t) is a function, which can be described as an infinite series (convergent in nature).

Literature has been enriched by work of numerous researchers who have contributed to field of Sumudu transformation. Sumudu transform concept was applied to PDEs by Kilicman and Gadain [45]. Via Sumudu transform method, Bulut et al. [46] provided analytical solution of fraction ODEs. Fundamental characteristics and uses of Sumudu transform were addressed by Belgacem and Karaballi [47]. To get an analytical solution to fractional ODEs, Demiray et al. [48] employed Sumudu transform method. Sumudu transform method and system of differential equations were addressed by Kilicman et al. [49]. Asiru [50] went into great length about Sumudu transform's characteristics as well as its uses. Regarding differential equations and Sumudu transform, Eltayeb and Kilicman [51] provided a note. Non-linear fractional PDEs were used to implement Sumudu HPM by Yousif and Hamed [52]. Via Table 1, basic properties regarding Sumudu transform are mentioned (see Table 2).

**Table 1:** Basic properties regarding Sumudu transform [47].

f(t)	S[f(t)]
1	1
t	θ
$\frac{t^{n-1}}{∠n-1}$, n=1,2,3,…	$\theta^{n-1}$
$e^{at}$	$\frac{1}{1-a\theta }$
$\frac{sinat}{a}$	$\frac{\theta }{1+a^{2}\theta^{2}}$
Cosat	$\frac{1}{1+a^{2}\theta^{2}}$
$\frac{sinhat}{a}$	$\frac{\theta }{1-a^{2}\theta^{2}}$
Coshat	$\frac{1}{1-a^{2}\theta^{2}}$

**Table 2:** Chart for Elzaki transform of the given function [53].

f(t)	E[f(t)]=T(ν)
1	$\nu^{2}$
t	$\nu^{3}$
$t^{n}$	$∠n\;\nu^{n+2}$
$e^{at}$	$\frac{\nu^{2}}{1-a\nu }$
sinat	$\frac{a\nu^{3}}{1+a^{2}\nu^{2}}$
cosat	$\frac{a\nu^{2}}{1+a^{2}\nu^{2}}$
sinhat	$\frac{a\nu^{3}}{1-a^{2}\nu^{2}}$
coshat	$\frac{a\nu^{2}}{1-a^{2}\nu^{2}}$

### 1.3 Elzaki transform

Basic notion of Elzaki transform is given as follows:

(1.6) $$E\left[f\left(t\right)\right]=\nu \int_{0}^{\infty }f\left(t\right)\exp\left(-\frac{t}{\nu }\right)dt,where\;t>0\text{.}$$

According to Tarig M. Elzaki and Salih M. Elzaki [[54], [55], [56]], modified Sumudu transform, also known as Elzaki transform, can be utilized to solve ODEs, PDEs, and systems of integral equations. Elzaki transform is a method which could be implemented effectively solve differential equations which cannot be solved via Sumudu transform. Elzaki transform in literature has been subject of extensive inquiry. By means of Elzaki HPM, Loyinmi and Akinfe [53] revealed precise solution of the Fisher's RD equation. ETM was applied to several FDEs by Khalid et al. [57]. Song and Kim [58] used ETM to provide solution for a VIE of the II sort. Regarding shifting theorem for ETM, Kim [59] provided a note.

Some properties regarding Elzaki transform are given as follows [53]:

(1.7) $$E\left[\frac{\partial }{\partial t}f\left(x,t\right)\right]=\frac{1}{\nu }\;T\left[x,\nu \right]-\nu f\left(x,0\right)$$

(1.8) $$E\left[\frac{\partial^{2}}{\partial t^{2}}f\left(x,t\right)\right]=\frac{1}{\nu^{2}}\;T\left[x,\nu \right]-f\left(x,0\right)-\nu \frac{\partial }{\partial t}f\left(x,0\right)\text{.}$$

(1.9) $$E\left[\frac{\partial }{\partial x}f\left(x,t\right)\right]=\frac{d}{dx}\left[T\left(x,\nu \right)\right]$$

(1.10) $$E\left[\frac{\partial^{2}}{\partial x^{2}}f\left(x,t\right)\right]=\frac{d^{2}}{dx^{2}}\left[T\left(x,\nu \right)\right]\text{.}$$

### 1.4 Homotopy perturbation method

The use of HPM for any non-linear differential equation is covered by He (1999) [60,61]. HPM was used by Biazar and Ghazvini [62] to provide accurate solutions to non-linear Schrodinger problem. To offer an exact solution to non-linear Burgers' equation, Biazar and Ghazvini [63] used HPM. The concept of a new HPM for solution of PDEs was introduced by Biazar and Eslami [64]. The HPM-based solution to KdV equation was presented by Yildirim [65]. The concept of HPM was used by Agirseven and Ozis [66] to provide an analytical solution of equations of Fisher's type. For analytical side of solving FPDEs, Yildirim [67] developed a conception of HPM.

The idea of sum of an infinite series term, which frequently converges quickly towards an exact solution, is connected to HPM. HPM reduces complex equations to a manageable, straightforward equation. Take into consideration following differential equation to define fundamental understanding of HPM.

(1.11) A(θ)=g(r),r∈D,

(1.12) $$with\;B\left(\theta ,\frac{\partial \theta }{\partial x}\right)=0,r\in \Gamma$$

where A is considered general operator, B is recognized as boundary operator, g(r) is considered as an analytic function, Γ is boundary of domain D. In general, operator A can be condensed into two operators, L and N, where L is linear operator and N is the non-linear operator. Equation (1.11)–(1.13) could be inscribed regarding basics of HPM;

(1.13) L(θ)+N(θ)=g(r).

Some latest work regarding analytical approximation of the differential equations is mentioned in literature [[68], [69], [70], [71], [72], [73]].

Novelty/Main Motivation/Advantages of the study.

The primary driving force behind this research is to identify exact and approximate analytical solutions to aforementioned coupled Burgers' equations. It is incredibly difficult to develop numerical programs that can solve PDEs of a complex type. To deal with such PDEs, it is therefore necessary to build such Integral transform-based approaches. Furthermore, discretization introduces a potential for inaccuracy in numerical techniques, but in suggested regime, discretization is not necessary. Because of this, such designed systems do not contain any discretization-related errors.

### 1.5 Limitations of the study

Despite being one of the most straightforward semi-analytical techniques to use, integral-transform based procedures. However, these regimes do have certain drawbacks, such as the inability to achieve a precise solution in some cases. Another shortcoming is the time-consuming calculation required by higher order fractional equations as the Sawada Kotera and KdV equations.

## 2 Outline of paper

The present paper is divided into different sections.

• In Section 1, a complete introduction of this research is notified. In Subsection 1.1, coupled 1D and 2D Burgers' equations are provided.
• In Subsection 1.2, concept of STM is introduced. In Subsection 1.3, concept of ETM is provided.
• In Subsection 1.4, a brief introduction of HPM is provided.
• In Section 3, Three Examples are discussed with aid of SHPM and EHPM, respectively. Example 1 and Example 2 are related to coupled 1D Burgers' equation and Example 3 is related to coupled 2D Burgers' equation. Numerical convergence aspect is also claimed.
• In Section 4, the crux of this comparison is concluded.

### 2.1 Convergence condition

Theorem 1 “Let F defined is an operator from Hilbert space. The series solution is considered as $u_{n}\left(x,t\right)=\sum_{i=0}^{n}u_{i}\left(x,t\right)$, which converges if there exists 0<η<1 such that,

$$\left|\left|F\left(u_{0}+u_{1}+u_{2}+u_{3}+\ldots +u_{i+1}\right)\right|\right|\leq k\;\left|\left|F\left(u_{0}+u_{1}+u_{2}+u_{3}+\ldots +u_{i}\right)\right|\right|\text{.}$$

for all i=0,1,2,3,… “

**Proof.** From Refs. [74,75].

Theorem 2 “If there is a series solution $u\left(x,t\right)=\sum_{i=0}^{\infty }u_{i}\left(x,t\right)$ considered as convergent, then the given series solution will denote the exact solution of the given linear/non-linear problem.”

**Proof.** From Refs. [74,75].

Theorem 3 “Considered the series solution $\sum_{i=0}^{\infty }u_{i}\left(x,t\right)$, is convergent to u(x,t), if the truncated series $\sum_{i=0}^{n}u_{i}\left(x,t\right)$ is considered as an approximate solution of the given problem then $\mathrm{Max}.\;Trunc.\;Error\leq \frac{1}{1-k}\;k\;\left|\left|u_{0}\right|\right|$.”

**Proof.** From Refs. [74,75].

In short, as per Theorem 1 and Theorem 2, solution obtained converges to the exact solution if 0<η<1 such that

$$\left|\left|F\left[u_{0}+u_{1}+u_{2}+u_{3}+\ldots +u_{i+1}\right]\right|\right|\leq \eta \left|\left|F\left[u_{0}+u_{1}+u_{2}+u_{3}+\ldots +u_{i+1}\right]\right|\right|\text{.}$$

i.e. $\left|\left|u_{i+1}\right|\right|\leq \eta \left|\left|u_{i}\right|\right|\text{.}$.

for all i=0,1,2,3,….

## 3 Examples and discussion

**Fig. 1:**
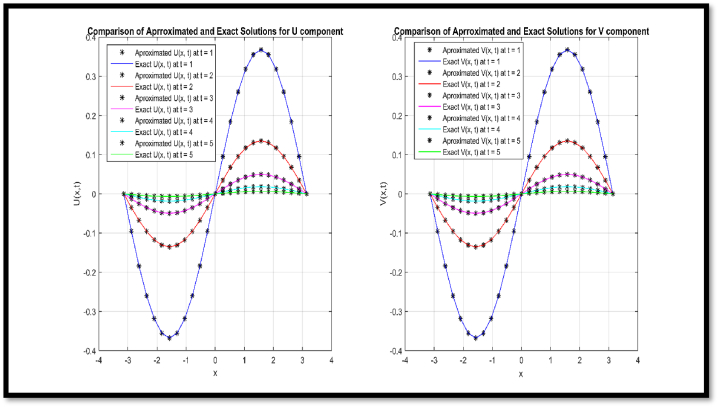
Comparison of Approximated and Exact solution of U(x,t) and V(x,t) for N = 25 at t = 1, 2, 3, 4 and 5 respectively.

In Fig. 1, a comparison of approx. and exact solution of θ(x,t) and η(x,t) for N = 25 at t = 1, 2, 3, 4, and 5 respectively is provided regarding Example 1. In Table 3, a comparison of approx. and exact θ and η components are provided for N = 11 at t = 0.1 and t = 1 regarding Example 1. In Table 4, a comparison of $L_{\infty }$ error for θ and η components are provided for N = 11 at diverse values of t regarding Example 1.

**Table 3:** Comparison of approximated and exact θ and η components for N = 11 at t = 0.1 and t = 1 regarding Example 1.

x	Approx. θ	Exact θ	Approx. η	Exact η
	t = 0.1			
0.6283	0.5319	0.5319	0.5319	0.5319
1.2566	0.8606	0.8606	0.8606	0.8606
1.885	0.8606	0.8606	0.8606	0.8606
	t = **1**			
0.6283	0.2162	0.2162	0.2162	0.2162
1.2566	0.3499	0.3499	0.3499	0.3499
1.885	0.3499	0.3499	0.3499	0.3499

**Table 4:** Comparison of $L_{\infty }$ error for θ and η components for N = 11 at diverse values of t regarding Example 1.

t	$L_{\infty }$θ	$L_{\infty }$η
0.1	1.1102E-16	1.1102E-16
0.2	4.4409eE-16	4.4409E-16
0.3	4.1189E-14	4.1189E-14
1	2.1983E-08	2.1983E-08
2	4.1757E-05	4.1757E-05
3	3.3656E-03	3.3656E-03

**Fig. 2:**
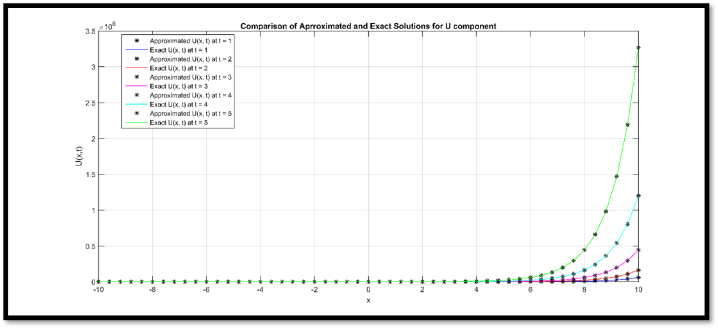
Comparison of Approximated and Exact solution of U(x,t) for N = 51 at t = 1, 2, 3, 4 and 5.

**Fig. 3:**
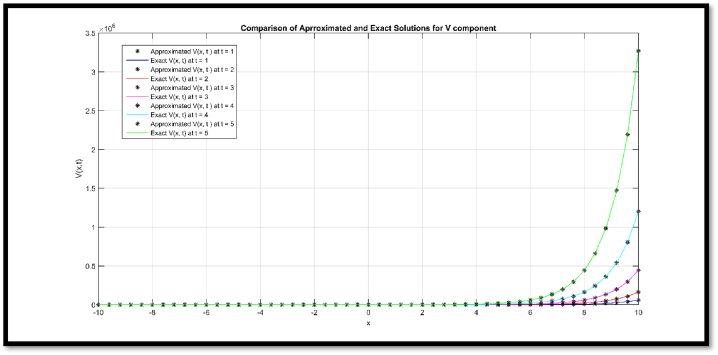
Comparison of Approximated and Exact solution of V(x,t) for N = 51 at t = 1, 2, 3, 4 and 5.

In Fig. 2, a comparison of approx. and exact solution of θ(x,t) for N = 51 at t = 1, 2, 3, 4 and 5 is provided regarding Example 2. In Fig. 3, comparison of approx. and exact solution of η(x,t) for N = 51 at t = 1, 2, 3, 4, and 5 regarding Example 2. In Table 6, a comparison of approx. and exact θ and η components are provided for N = 11 at t = 0.1 and t = 1 regarding Example 2. In Table 7, a comparison of $L_{\infty }$ error for θ and η components are provided for N = 11 at diverse values of t regarding Example 2.

**Table 5:** Numerical convergence aspect for θ and η components regarding Example 1.

N	$L_{\infty }$	$L_{\infty }$	$L_{\infty }$
	t = 1	t = 2	t = 3
10	2.4862E-07	2.3466E-04	1.2540E-02
20	5.5511E-17	3.9199E-13	1.2488E-09
30	1.6653E-16	1.1102E-16	4.8572E-16
	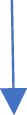 Converging up to $10^{-16}$	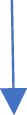 Converging up to $10^{-16}$	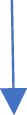 Converging up to $10^{-16}$

**Table 6:** Comparison of approximated and exact θ and η components for N = 11 at t = 0.1 and t = 1 regarding Example 2.

x	Approx. θ	Exact θ	Approx. η	Exact η
t = 0.1				
−10	5.02E-05	5.02E-05	5.02E-05	5.02E-05
−8	3.71E-04	3.71E-04	3.71E-04	3.71E-04
−6	2.74E-03	2.74E-03	2.74E-03	2.74E-03
t = **1**				
−10	1.23E-04	1.23E-04	1.23E-04	1.23E-04
−8	9.12E-04	9.12E-04	9.12E-04	9.12E-04
−6	6.74E-03	6.74E-03	6.74E-03	6.74E-03

**Table 7:** Comparison of $L_{\infty }$ error for θ and η components for N = 11 at diverse values of t regarding Example 2.

t	$L_{\infty }$θ	$L_{\infty }$η
**0.1**	4.5475E-13	4.5475E-13
**0.2**	1.4552E-11	1.4552E-11
**0.3**	1.0041E-09	1.0041E-09

**Fig. 4:**
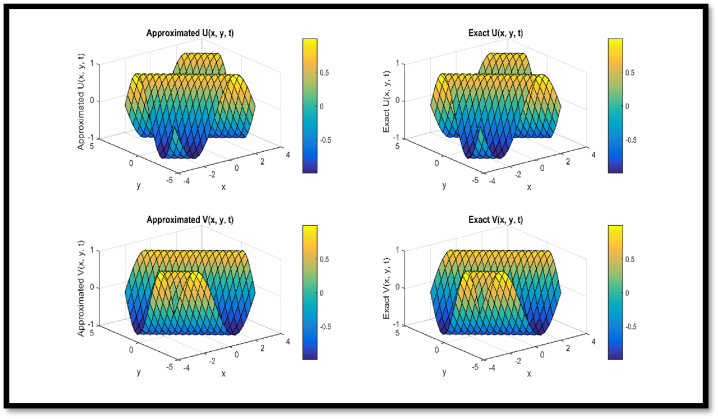
Comparison of Approximated and Exact solutions for U and V components for N = 21 at t = 0.1.

**Fig. 5:**
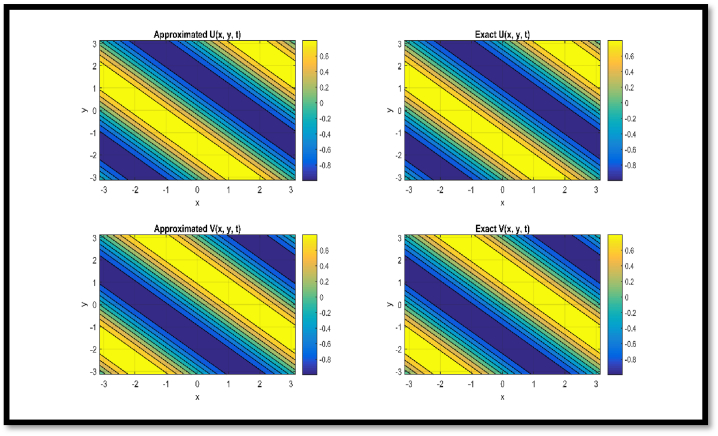
Contour representation for comparison of Approximated and Exact solutions for U and V components for N = 21 at t = 0.1.

**Fig. 6:**
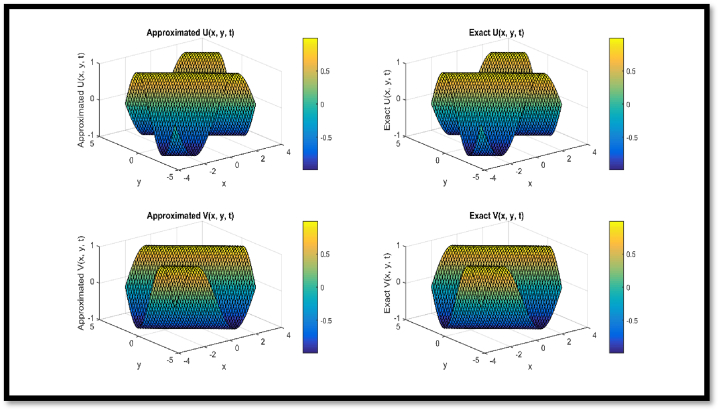
Comparison of Approximated and Exact solutions for U and V components for N = 51 at t = 1.

**Fig. 7:**
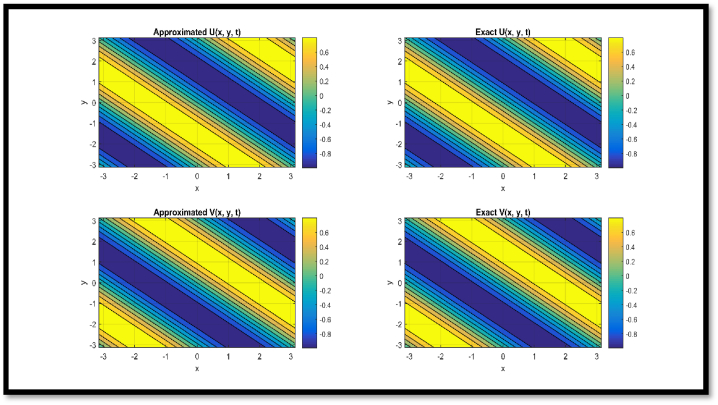
Contour representation for comparison of Approximated and Exact solutions for U and V components for N = 51 at t = 1.

In Fig. 4, a comparison of approx. and exact solutions for θ and η components for N = 21 at t = 0.1 is provided regarding Example 3. In Fig. 5, contour representation for comparison of approx. and exact solutions for θ and η components for N = 21 at t = 0.1 is provided regarding Example 3. In Fig. 6, a comparison of approx. and exact solutions for θ and η components for N = 51 at t = 1 regarding Example 3. In Fig. 7, contour representation for comparison of approx. and exact solutions for θ and η components for N = 51 at t = 1 is provided regarding Example 3. Considered θ=U and η=V in pictorial depiction. Table 9, a comparison of approx. and exact θ and η components is provided for N = 10, ν = $\frac{1}{500}$ at t = 0.1 and t = 1 regarding Example 3. Table 10, Comparison of $L_{\infty }$ error for θ and η components is provided for N = 10, ν = $\frac{1}{500}$ at diverse values of t regarding Example 3.

**Table 8:** Numerical convergence aspect for θ and η components regarding Example 2.

N	$L_{\infty }$	$L_{\infty }$	$L_{\infty }$
	t = 1	t = 1.3	t = 1.5
**10**	6.6715E-03	9.4757E-02	4.0449E-01
**15**	1.7964E-08	9.3802E-07	8.1339E-06
**20**	3.6380E-12	1.4552E-11	1.4552E-11
	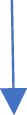 Converging up to $10^{-12}$	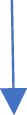 Converging up to $10^{-11}$	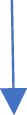 Converging up to $10^{-11}$

**Table 9:** Comparison of approximated and exact θ and η components for N = 10, ν = $\frac{1}{500}$ at t = 0.1 and t = 1 regarding Example 3.

(x,y)	Approx. θ	Exact θ	Approx. η	Exact η
	t = 0.1			
**(1.05, 1.04)**	−0.86568	−0.86568	0.865679	0.865679
**(1.75, 1.74)**	0.341883	0.341883	−0.34188	−0.34188
**(2.44, 2.44)**	0.984414	0.984414	−0.98441	−0.98441
	t = **1**			
**(1.05, 1.04)**	−0.86257	−0.86257	0.862568	0.862568
**(1.75, 1.74)**	0.340655	0.340655	−0.34065	−0.34065
**(2.44, 2.44)**	0.980876	0.980876	−0.98088	−0.98088

**Table 10:** Comparison of $L_{\infty }$ error for θ and η components for N = 10, ν = $\frac{1}{500}$ at diverse values of t regarding Example 3.

t	$L_{\infty }$θ	$L_{\infty }$η
**0.1**	1.1102E-16	1.1102E-16
**0.2**	4.9304E-32	4.9304E-32
**0.3**	2.2204E-16	2.2204E-16
**1**	1.1102E-16	1.1102E-16
**2**	1.1102E-16	1.1102E-16
**3**	1.1102E-16	1.1102E-16

Via Fig. 1, compatibility of approx. and exact solutions is affirmed at a wide range of time levels for Example 1. Via Fig. 2, Fig. 3, approx. and exact solutions are matched for compatibility at a vast range of time levels for Example 2. In Figs. 4–7, via surface and contour graphs are provided for approx. and exact solutions regarding Example 3.

Via Table 5 and Table 8, aspect of numerical convergence is also claimed for the proposed regime. It is noticed that via Table 5, Table 8, that on increasing number of grid points the $L_{\infty }$ error got reduced, which is an assurance of numerical convergence. Via Tables 5 and it is notified that at t = 1, 2, and 3, error got reduced up to $10^{-16}$. Via Tables 8 and it is notified that at t = 1, error is reduced up to $10^{-12}$, at t = 1.3, error got reduced up to $10^{-11}$, and at t = 1.5, error got reduced up to $10^{-11}$.

Example 1 In this example, coupled 1D Burgers’ equation is considered as follows [1,2]:

(2.1) $$\theta_{t}-\theta_{xx}-2\;\theta \;\theta_{x}+{\left(\theta V\right)}_{x}=0\text{,}$$

(2.2) $$\eta_{t}-\eta_{xx}-2\;\eta \;\eta_{x}+{\left(\theta \eta \right)}_{x}=0\text{.}$$

I.C.s:θ(x,0)=sinx,η(x,0)=sinx.

Domain of computation = [−π,π].

From Equation (2.1), Equation (2.3) can be notified as follows:

(2.3) $$\theta_{t}=\theta_{xx}+2\;\theta \;\theta_{x}-{\left(\theta \eta \right)}_{x}\text{,}$$

(2.4) $$\Rightarrow \;\theta_{t}=\theta_{xx}+2\;\theta \;\theta_{x}-\left[\theta \;\eta_{x}+\eta \;\theta_{x}\right]\text{.}$$

• Application of Sumudu HPM [Method I]

Applying Sumudu transform in Equation (2.4), Equations (2.5)–(2.7) can be updated as follows:

(2.5) $$S\left[\theta_{t}\right]=S\left[\theta_{xx}+2\;\theta \;\theta_{x}-\left\{\theta \eta_{x}+\eta \;\theta_{x}\right\}\right]\text{,}$$

(2.6) $$\Rightarrow \;1/\theta \;\left[S\left(\theta \left(x,t\right)\right)-\theta \left(x,0\right)\right]=S\left[\theta_{xx}+2\;\theta \;\theta_{x}-\left\{\theta \eta_{x}+\eta \;\theta_{x}\right\}\right]\text{,}$$

(2.7) $$\Rightarrow \;S\left(\theta \left(x,t\right)\right)=\theta \left(x,0\right)+\theta \;S\left[\theta_{xx}+2\;\theta \;\theta_{x}-\left\{\theta \eta_{x}+\eta \;\theta_{x}\right\}\right]\text{,}$$

(2.8) $$\Rightarrow \theta \left(x,t\right)=sinx+S^{-1}\left[\theta \;S\left[\theta_{xx}+2\;\theta \;\theta_{x}-\left\{\theta \eta_{x}+\eta \;\theta_{x}\right\}\right]\right]\text{.}$$

By implementing HPM in Equation (2.8), Equation (2.9) will be notified as follows:

(2.9) $$\Rightarrow \sum_{n=0}^{\infty }p^{n}\theta_{n}\left(x,t\right)=sinx+pS^{-1}\left[\theta \;S\left[{\left\{\sum_{n=0}^{\infty }p^{n}\theta_{n}\left(x,t\right)\right\}}_{xx}+2\;\theta \;\theta_{x}-\left\{\theta \eta_{x}+\eta \;\theta_{x}\right\}\right]\right]$$

(2.10) $$\Rightarrow \sum_{n=0}^{\infty }p^{n}\theta_{n}\left(x,t\right)=sinx+pS^{-1}\left[\theta \;S\left[{\left\{\sum_{n=0}^{\infty }p^{n}\theta_{n}\left(x,t\right)\right\}}_{xx}+\sum_{n=0}^{\infty }p^{n}H_{n}\left(\theta ,\eta \right)\right]\right]$$

Where $H_{n}\left(\theta ,\eta \right)=2\;\theta \;\theta_{x}-\left\{\theta \eta_{x}+\eta \;\theta_{x}\right\}$. Where $H_{n}\left(\theta ,\eta \right)$ is representing the non-linear term.

In Equation (2.11), the non-linear term is notified.

(2.11) $$H_{0}\left(\theta ,\eta \right)=2\;\theta_{0}\;{\left(\theta_{0}\right)}_{x}-\left\{\theta_{0}\;{\left(\eta_{0}\right)}_{x}+\eta_{0}\;{\left(\theta_{0}\right)}_{x}\right\}\text{.}$$

From Equation (2.10), comparing powers of p:

(2.12) $$p^{0}:\theta_{0}\left(x,t\right)=sinx\text{.}$$

Via Equation (2.12), power of $p^{0}$ is compared.

$$p^{1}:\theta_{1}\left(x,t\right)=S^{-1}\left[\theta S\left\{{\left(\theta_{0}\right)}_{xx}+H_{0}\left(\theta \right)\right\}\right]\text{,}$$

$$=S^{-1}\left[\theta \;S\left\{-sinx\right\}\right]\text{,}$$

$$=\left(-sinx\right)S^{-1}\left[\theta \right]\text{,}$$

(2.13) $$\Rightarrow \theta_{1}\left(x,t\right)=-sinx\;t$$

Via Equation (2.13), second term of the series approximation is fetched.

(2.14) $$p^{2}:\theta_{2}\left(x,t\right)=S^{-1}\left[\theta S\left\{{\left(\theta_{1}\right)}_{xx}+H_{1}\left(\theta \right)\right\}\right]\text{.}$$

where,

$$H_{1}\left(\theta ,\eta \right)=2\;\left[\theta_{1}{\left(\theta_{0}\right)}_{x}+\theta_{0}\;{\left(\theta_{1}\right)}_{x}\right]-\left[\theta_{1}{\left(\eta_{0}\right)}_{x}+\theta_{0}\;{\left(\eta_{1}\right)}_{x}+{\left(\theta_{1}\right)}_{x}\;\eta_{0}+{\left(\theta_{0}\right)}_{x}\eta_{1}\right]\text{,}$$

$$\Rightarrow H_{1}\left(\theta ,\eta \right)=2\;\left[-sinx\;t\;cosx+sinx\;\left(-cosx\;t\right)\right]-\left[\left(-sinx\right)t\;cosx+sinx\;\left(-cosx\;t\right)+\left(-cosx\;t\;sinx\right)+cosx\;\left(-sinx\;t\right)\right]\text{,}$$

$$\Rightarrow H_{1}\left(\theta ,\eta \right)=2\;\left(-2\;sinx\;cosx\;t\right)-\left[-4\;sinx\;cosx\;t\right]\text{,}$$

$$\Rightarrow H_{1}\left(\theta ,\eta \right)=0\text{.}$$

Using $H_{1}\left(\theta ,\eta \right)$ in Equation (2.14):

$$\theta_{2}\left(x,t\right)=S^{-1}\left[\theta S\left\{{\left(\theta_{1}\right)}_{xx}\right\}\right]\text{,}$$

$$\Rightarrow \theta_{2}\left(x,t\right)=S^{-1}\left[\theta \;S\left\{\left(sinx\right)t\right\}\right]\;\Rightarrow \theta_{2}\left(x,t\right)=S^{-1}\left[\theta \left(sinx\right)\;S\left(t\right)\}\right]\text{,}$$

$$\Rightarrow \theta_{2}\left(x,t\right)=S^{-1}\left[\theta \left(sinx\right)\theta \}\right]\;\Rightarrow \theta_{2}\left(x,t\right)={\left(sinx\right)\;S}^{-1}\left[\theta^{2}\}\right]\text{,}$$

$$\Rightarrow \theta_{2}\left(x,t\right)=\frac{\left(sinx\right)t^{2}}{2}\text{.}$$

In a general way, $\theta \left(x,t\right)=\theta_{0}\left(x,t\right)+\theta_{1}\left(x,t\right)+\theta_{2}\left(x,t\right)+\ldots$.

$$\Rightarrow \theta \left(x,t\right)=sinx-sinx\;t+sinx\frac{t^{2}}{2}-\ldots \;\Rightarrow \theta \left(x,t\right)=sinx\;\left[1-t+\frac{t^{2}}{2}-\ldots \right]$$

(2.15) ⇒θ(x,t)=exp(−t)sinx.

Via Equation (2.15), the exact solution for θ component is provided.

From Equation (2.2), Equation (2.16) is updated as follows:

(2.16) $$\eta_{t}-\eta_{xx}-2\;\eta \;\eta_{x}+{\left(\theta \eta \right)}_{x}=0\text{,}$$

(2.17) $$\eta_{t}=\eta_{xx}+2\;\eta \;\eta_{x}-{\left(\theta \eta \right)}_{x}\text{,}$$

Applying Sumudu transform in Equation (2.17), Equations (2.18)–(2.31) will be updated as:

(2.18) $$S\left[\eta_{t}\right]=S\left[\eta_{xx}+2\;\eta \;\eta_{x}-\left\{\theta \eta_{x}+\eta \;\theta_{x}\right\}\right]\text{,}$$

(2.19) $$\frac{1}{\eta }\;\left[S\left(\eta \left(x,t\right)\right)-\eta \left(x,0\right)\right]=S\left[\eta_{xx}+2\;\eta \;\eta_{x}-\left\{\theta \eta_{x}+\eta \;\theta_{x}\right\}\right]\text{,}$$

(2.20) $$S\left(\eta \left(x,t\right)\right)=\eta \left(x,0\right)+\eta \;S\left[\eta_{xx}+2\;\eta \;\eta_{x}-\left\{\theta \eta_{x}+\eta \;\theta_{x}\right\}\right]\text{,}$$

(2.21) $$\eta \left(x,t\right)=sinx+S^{-1}\left[\eta \;S\left[\eta_{xx}+2\;\eta \;\eta_{x}-\left\{\theta \eta_{x}+\eta \;\theta_{x}\right\}\right]\right]\text{.}$$

By implementing HPM in Equation number (2.21), Equation (2.22) will be notified as:

(2.22) $$\Rightarrow \sum_{n=0}^{\infty }p^{n}\eta_{n}\left(x,t\right)=sinx+pS^{-1}\left[\eta \;S\left[{\left\{\sum_{n=0}^{\infty }p^{n}\eta_{n}\left(x,t\right)\right\}}_{xx}+2\;\eta \;\eta_{x}-\left\{\theta \eta_{x}+\eta \;\theta_{x}\right\}\right]\right]\text{,}$$

(2.23) $$\sum_{n=0}^{\infty }p^{n}\eta_{n}\left(x,t\right)=sinx+pS^{-1}\left[\eta \;S\left[{\left\{\sum_{n=0}^{\infty }p^{n}\eta_{n}\left(x,t\right)\right\}}_{xx}+\sum_{n=0}^{\infty }p^{n}I_{n}\left(\theta ,\eta \right)\right]\right]\text{,}$$

Where $I_{n}\left(\theta ,\eta \right)=2\;\eta \;\eta_{x}-\left\{\theta \eta_{x}+\eta \;\theta_{x}\right\}$, Where $I_{n}\left(\theta ,\eta \right)$ is representing the non-linear term.

Equation (2.24) is provided regarding the non-linear term.

(2.24) $$I_{0}\left(\theta ,\eta \right)=2\;\eta_{0}\;{\left(\eta_{0}\right)}_{x}-\left\{\theta_{0}\;{\left(\eta_{0}\right)}_{x}+\eta_{0}\;{\left(\theta_{0}\right)}_{x}\right\}\text{.}$$

From Equation (2.23), on comparing powers of p:

(2.25) $$p^{0}:\eta_{0}\left(x,t\right)=sinx\text{.}$$

Via Equation (2.25), first term of the series solution is notified.

$$p^{1}:\eta_{1}\left(x,t\right)=S^{-1}\left[\eta S\left\{{\left(V_{0}\right)}_{xx}+I_{0}\left(\theta ,\eta \right)\right\}\right]\text{,}$$

$$\Rightarrow \eta_{1}\left(x,t\right)=S^{-1}\left[\eta \;S\left\{-sinx\right\}\right]\text{,}$$

$$\Rightarrow \eta_{1}\left(x,t\right)=\left(-sinx\right)S^{-1}\left[\eta \right]\text{,}$$

(2.26) $$\Rightarrow \eta_{1}\left(x,t\right)=-\left(sinx\right)\;t$$

Via Equation (2.26), the second term of the series solution is provided.

(2.27) $$p^{2}:\eta_{2}\left(x,t\right)=S^{-1}\left[\eta \;S\left\{{\left(\eta_{1}\right)}_{xx}+I_{1}\left(\theta ,\eta \right)\right\}\right]$$

where,

$$I_{1}\left(\theta ,\eta \right)=2\;\left[\eta_{1}{\left(\eta_{0}\right)}_{x}+\eta_{0}\;{\left(\eta_{1}\right)}_{x}\right]-\left[\theta_{1}{\left(\eta_{0}\right)}_{x}+\theta_{0}\;{\left(\eta_{1}\right)}_{x}+{\left(\theta_{1}\right)}_{x}\;\eta_{0}+{\left(\theta_{0}\right)}_{x}\eta_{1}\right]$$

$$\Rightarrow I_{1}\left(\theta ,\eta \right)=0\text{.}$$

Using $I_{1}\left(\theta ,\eta \right)$ = 0 in Equation (2.27):

$$\eta_{2}\left(x,t\right)=S^{-1}\left[\eta S\left\{{\left(\eta_{1}\right)}_{xx}\right\}\right]\Rightarrow \eta_{2}\left(x,t\right)=S^{-1}\left[\eta \;S\left\{\left(sinx\right)t\right\}\right]\text{,}$$

$$\Rightarrow \eta_{2}\left(x,t\right)=S^{-1}\left[\eta \left(sinx\right)\eta \}\right]\Rightarrow \eta_{2}\left(x,t\right)={\left(sinx\right)S}^{-1}\left[\eta^{2}\}\right]\text{,}$$

(2.28) $$\Rightarrow \eta_{2}\left(x,t\right)=\frac{\left(sinx\right)t^{2}}{2}$$

in Equation (2.28), the third term of the series solution if obtained.

In a general way,

$$\eta \left(x,t\right)=\eta_{0}\left(x,t\right)+\eta_{1}\left(x,t\right)+\eta_{2}\left(x,t\right)+\ldots$$

$$\Rightarrow \eta \left(x,t\right)=sinx-sinx\;t+sinx\frac{t^{2}}{2}-\ldots$$

$$\Rightarrow \eta \left(x,t\right)=sinx\;\left[1-t+\frac{t^{2}}{2}-\ldots \right]\text{,}$$

(2.29) ⇒η(x,t)=exp(−t)sinx.

Exact solutions in this example are provided as follows:

(2.30) θ(x,t)=exp(−t)sinx,

(2.31) η(x,t)=exp(−t)sinx.

Via Equations (2.29)–(2.31), exact solutions are provided.

• Application of Elzaki HPM [Method II]

Applying Elzaki transform on Equation (2.4):

$$E[\theta_{t}]={E[\theta }_{xx}+2\;\theta \;\theta_{x}-{\left(\theta \eta \right)}_{x}]\text{,}$$

$$\frac{1}{\nu }\;E\left[\theta \left(x,t\right)\right]-\nu \theta \left(x,0\right)={E[\theta }_{xx}+2\;\theta \;\theta_{x}-{\left(\theta \eta \right)}_{x}]\text{,}$$

$$\Rightarrow \frac{1}{\nu }\;E\left[\theta \left(x,t\right)\right]=\nu \;\theta \left(x,0\right)+{E[\theta }_{xx}+2\;\theta \;\theta_{x}-{\left(\theta \eta \right)}_{x}]\text{,}$$

$$\Rightarrow E\left[\theta \left(x,t\right)\right]=\nu^{2}\theta \left(x,0\right)+\nu {E[\theta }_{xx}+2\;\theta \;\theta_{x}-{\left(\theta \eta \right)}_{x}]\text{,}$$

$$\Rightarrow \theta \left(x,t\right)=\theta \left(x,0\right)E^{-1}\left(\nu^{2}\right)+E^{-1}[\nu {E[\theta }_{xx}+2\;\theta \;\theta_{x}-{\left(\theta \eta \right)}_{x}]]\text{,}$$

(2.32) $$\Rightarrow \theta \left(x,t\right)=sinx+E^{-1}[\nu {E[\theta }_{xx}+2\;\theta \;\theta_{x}-{\left(\theta \eta \right)}_{x}]]\text{.}$$

Applying HPM in Equation (2.32):

(2.33) $$\sum_{n=0}^{\infty }p^{n}\theta_{n}\left(x,t\right)=sinx+p\;E^{-1}\left[\nu E\left\{{\left(\sum_{n=0}^{\infty }p^{n}\theta_{n}\left(x,t\right)\right)}_{xx}+\sum_{n=0}^{\infty }p^{n}H_{n}\left(\theta ,\eta \right)\right\}\right]\text{.}$$

Where, $H_{n}\left(\theta ,\eta \right)=2\;\theta \;\theta_{x}-{\left(\theta \eta \right)}_{x}$, $H_{n}\left(\theta ,\eta \right)$ is a non-linear term.

$$H_{0}\left(\theta ,\eta \right)=2\;\theta_{0}{\left(\theta_{0}\right)}_{x}-\left[\theta_{0}{\left(\eta_{0}\right)}_{x}+{\left(\theta_{0}\right)}_{x}\eta_{0}\right]\text{,}$$

$$H_{1}\left(\theta ,\eta \right)=2\left[\theta_{1}{\left(\theta_{0}\right)}_{x}+\theta_{0}{\left(\theta_{1}\right)}_{x}\right]-\left[\theta_{1}{\left(\eta_{0}\right)}_{x}+\theta_{0}{\left(\eta_{1}\right)}_{x}+{\left(\theta_{1}\right)}_{x}\eta_{0}+{\left(\theta_{0}\right)}_{x}\eta_{1}\right]\text{,}$$

Comparing powers of p in Equation (2.33):

$p^{0}:\theta_{0}\left(x,t\right)=sinx$.

$$p^{1}:\theta_{1}\left(x,t\right)=E^{-1}\left[\nu E\left\{{\left(\theta_{0}\left(x,t\right)\right)}_{xx}+H_{0}\left(\theta ,\eta \right)\right\}\right]\text{,}$$

$$\theta_{1}\left(x,t\right)=E^{-1}\left[\nu E\left\{-sinx\right\}\right]\text{,}$$

$$\Rightarrow \theta_{1}\left(x,t\right)=-sinx\;E^{-1}\left[\nu E\left(1\right)\right]\text{,}$$

$$\Rightarrow \theta_{1}\left(x,t\right)=-sinx\;E^{-1}\left[\nu^{3}\right]\text{,}$$

$$\Rightarrow \theta_{1}\left(x,t\right)=-t\;sinx\text{.}$$

$$p^{2}:\theta_{2}\left(x,t\right)=E^{-1}\left[\nu E\left\{{\left(\theta_{1}\left(x,t\right)\right)}_{xx}+H_{1}\left(\theta ,\eta \right)\right\}\right]\text{,}$$

$$\theta_{2}\left(x,t\right)=E^{-1}\left[\nu E\left\{t\;sinx\right\}\right]\Rightarrow \theta_{2}\left(x,t\right)=sinxE^{-1}\left[\nu E\left\{t\right\}\right]\text{,}$$

$$\Rightarrow \theta_{2}\left(x,t\right)=sinxE^{-1}\left[\nu \;\nu^{3}\right]\Rightarrow \theta_{2}\left(x,t\right)=sinxE^{-1}\left[\nu^{4}\right]\text{,}$$

$$\Rightarrow \theta_{2}\left(x,t\right)=sinx\;\left(\frac{t^{2}}{∠2}\right)$$

$$\theta \left(x,t\right)=\theta_{0}\left(x,t\right)+\theta_{1}\left(x,t\right)+\theta_{2}\left(x,t\right)+\ldots$$

$$\Rightarrow \theta \left(x,t\right)=sinx-t\;sinx+sinx\;\left(\frac{t^{2}}{∠2}\right)-\ldots$$

(2.34) θ(x,t)=exp(−t)sinx

in Equation (2.34), exact solution is provided.

Applying Elzaki transform on Equation (2.2):

$$E[\eta_{t}]={E[\eta }_{xx}+2\;\eta \;\eta_{x}-{\left(\theta \eta \right)}_{x}]\text{,}$$

$$\frac{1}{\nu }\;E\left[\eta \left(x,t\right)\right]-\nu \;\eta \left(x,0\right)={E[\eta }_{xx}+2\;\eta \;\eta_{x}-{\left(\theta \eta \right)}_{x}]\text{,}$$

$$\Rightarrow \frac{1}{\nu }\;E\left[\eta \left(x,t\right)\right]=\nu \;\eta \left(x,0\right)+{E[\eta }_{xx}+2\;\eta \;\eta_{x}-{\left(\theta \eta \right)}_{x}]\text{,}$$

$$\Rightarrow E\left[\eta \left(x,t\right)\right]=\nu^{2}\;\eta \left(x,0\right)+\nu \;{E[\eta }_{xx}+2\;\eta \;\eta_{x}-{\left(\theta \eta \right)}_{x}]\text{,}$$

$$\Rightarrow \eta \left(x,t\right)=\eta \left(x,0\right)E^{-1}\left(\nu^{2}\right)+E^{-1}[\nu {E[\eta }_{xx}+2\;\eta \;\eta_{x}-{\left(\theta \eta \right)}_{x}]]\text{,}$$

(2.35) $$\Rightarrow \eta \left(x,t\right)=sinx+E^{-1}[\nu \;{E[\eta }_{xx}+2\;\eta \;\eta_{x}-{\left(\theta \eta \right)}_{x}]]\text{.}$$

Applying HPM in Equation (2.35):

$$\sum_{n=0}^{\infty }p^{n}\eta_{n}\left(x,t\right)=sinx+p\;E^{-1}\left[\nu E\left\{{\left(\sum_{n=0}^{\infty }p^{n}\eta_{n}\left(x,t\right)\right)}_{xx}+\sum_{n=0}^{\infty }p^{n}I_{n}\left(\theta ,\eta \right)\right\}\right]\text{.}$$

Where, $I_{n}\left(\theta ,\eta \right)=2\;\eta \eta_{x}-{\left(\theta \eta \right)}_{x}$ [non-linear term]

$$I_{0}\left(\theta ,\eta \right)=2\;\eta_{0}{\left(\eta_{0}\right)}_{x}-\left[\theta_{0}{\left(\eta_{0}\right)}_{x}+{\left(\theta_{0}\right)}_{x}\eta_{0}\right]\text{.}$$

Comparing powers of p:

$$p^{0}:\eta_{0}\left(x,t\right)=sinx\text{.}$$

$$p^{1}:\eta_{1}\left(x,t\right)=E^{-1}\left[\nu E\left\{{\left(\eta_{0}\left(x,t\right)\right)}_{xx}+I_{0}\left(\theta ,\eta \right)\right\}\right]\text{,}$$

$$\eta_{1}\left(x,t\right)=E^{-1}\left[\nu E\left\{-sinx\right\}\right]\Rightarrow \eta_{1}\left(x,t\right)=-sinx\;E^{-1}\left[\nu E\left(1\right)\right]\text{,}$$

$$\Rightarrow \eta_{1}\left(x,t\right)=-sinx\;E^{-1}\left[\nu^{3}\right]\Rightarrow \eta_{1}\left(x,t\right)=-t\;sinx\text{.}$$

$$p^{2}:\eta_{2}\left(x,t\right)=E^{-1}\left[\nu E\left\{{\left(V_{1}\left(x,t\right)\right)}_{xx}+I_{1}\left(\theta ,V\right)\right\}\right]\text{,}$$

$$\eta_{2}\left(x,t\right)=E^{-1}\left[\nu E\left\{t\;sinx\right\}\right]\Rightarrow \eta_{2}\left(x,t\right)=sinxE^{-1}\left[\nu E\left\{t\right\}\right]\text{,}$$

$$\Rightarrow \eta_{2}\left(x,t\right)=sinxE^{-1}\left[\nu \;\nu^{3}\right]\Rightarrow \eta_{2}\left(x,t\right)=sinxE^{-1}\left[\nu^{4}\right]\text{,}$$

$$\eta_{2}\left(x,t\right)=sinx\;\left(\frac{t^{2}}{∠2}\right)\text{.}$$

$$\eta \left(x,t\right)=\eta_{0}\left(x,t\right)+\eta_{1}\left(x,t\right)+\eta_{2}\left(x,t\right)+\ldots$$

$$\eta \left(x,t\right)=sinx-t\;sinx+sinx\;\left(\frac{t^{2}}{∠2}\right)-\ldots$$

η(x,t)=exp(−t)sinx.

Exact solutions are as follows:

θ(x,t)=exp(−t)sinxandη(x,t)=exp(−t)sinx.

### 3.1 Convergence analysis

$\mu_{0}=\frac{\left\|\theta_{1}\right\|}{\left\|\theta_{0}\right\|}$ = 1.0000E-02 < 1, $\mu_{1}=\frac{\left\|\theta_{2}\right\|}{\left\|\theta_{1}\right\|}$ = 5.0000E-03 < 1

$\mu_{2}=\frac{\left\|\theta_{3}\right\|}{\left\|\theta_{2}\right\|}$ = 3.3333E-03 < 1, $\mu_{3}=\frac{\left\|\theta_{4}\right\|}{\left\|\theta_{3}\right\|}$ = 2.5000E-03 < 1

And $\nu_{0}=\frac{\left\|\eta_{1}\right\|}{\left\|\eta_{0}\right\|}$ = 1.0000E-02 < 1, $\nu_{1}=\frac{\left\|\eta_{2}\right\|}{\left\|\eta_{1}\right\|}$ = 5.0000E-03 < 1

$\nu_{2}=\frac{\left\|\eta_{3}\right\|}{\left\|\eta_{2}\right\|}$ = 3.3333E-03 < 1, $\nu_{3}=\frac{\left\|\eta_{4}\right\|}{\left\|\eta_{3}\right\|}$ = 2.5000E-03 < 1

$\mu_{i}^{\prime }s$ and ${\nu_{i}}^{\prime }s$ for i≥0 and $t\leq \left(\frac{j}{2}\right)$ for 0<j<1 are less than one. Hence convergence condition is completed.

Example 2 Via Equations (2.36)–(2.37) coupled 1D Burgers’ equations are notified.

In this example, coupled 1D Burgers’ equation is considered as follows [2]:

(2.36) $$\theta_{t}+2\;\theta \;\theta_{x}-{\left[\theta V\right]}_{x}=\theta_{xx}\text{,}$$

(2.37) $$\eta_{t}+2\;\eta \;\eta_{x}-{\left[\theta \eta \right]}_{x}=\eta_{xx}\text{.}$$

(2.38) $$\theta_{t}=\theta_{xx}-2\;\theta \;\theta_{x}+{\left[\theta \eta \right]}_{x}\text{,}$$

(2.39) $$\eta_{t}=\eta_{xx}-2\;\eta \;\eta_{x}+{\left[\theta \eta \right]}_{x}$$

**Initial conditions:**$\theta \left(x,0\right)=e^{x}$, $\eta \left(x,0\right)=e^{x}$.

• Application of Sumudu HPM [Method I]

On applying Sumudu transform on Equation (2.38):

$${S[\theta }_{t}]={S[\theta }_{xx}-2\;\theta \;\theta_{x}+{\left[\theta \eta \right]}_{x}]\text{,}$$

$$\Rightarrow \frac{1}{\theta }\left[S\left(\theta \left(x,t\right)\right)-\theta \left(x,0\right)\right]={S[\theta }_{xx}-2\;\theta \;\theta_{x}+{\left[\theta \eta \right]}_{x}]\text{,}$$

$$\Rightarrow \left[S\left(\theta \left(x,t\right)\right)-\theta \left(x,0\right)\right]=\theta [{S[\theta }_{xx}-2\;\theta \;\theta_{x}+{\left[\theta \eta \right]}_{x}]]\text{,}$$

$$\Rightarrow \left[S\left(\theta \left(x,t\right)\right)\right]=\theta \left(x,0\right)+\theta \left[{S[\theta }_{xx}-2\;\theta \;\theta_{x}+{\left[\theta \eta \right]}_{x}\right]\text{,}$$

$$\Rightarrow \left[S\left(\theta \left(x,t\right)\right)\right]=e^{x}+\theta \left[{S[\theta }_{xx}-2\;\theta \;\theta_{x}+{\left[\theta \eta \right]}_{x}\right]\text{,}$$

(2.40) $$\theta \left(x,t\right)=e^{x}+S^{-1}\left[\theta \left[{S[\theta }_{xx}-2\;\theta \;\theta_{x}+{\left[\theta \eta \right]}_{x}\right]\right]\text{.}$$

where, $H_{n}\left(\theta ,\eta \right)={\left(\theta \eta \right)}_{x}-2\;\theta \;\theta_{x}$ , $H_{n}\left(\theta ,\eta \right)$ is representing non-linear term.

$$\Rightarrow H_{n}\left(\theta ,\eta \right)=\left(\theta_{x}\eta +\theta \;\eta_{x}\right)-2\;\theta \;\theta_{x}\text{.}$$

By applying HPM in Equation (2.40), Equation (2.41) will be notified as follows:

(2.41) $$\Rightarrow \sum_{n=0}^{\infty }p^{n}\theta_{n}\left(x,t\right)=e^{x}+pS^{-1}\left[\theta \;S\left[{\left\{\sum_{n=0}^{\infty }p^{n}\theta_{n}\left(x,t\right)\right\}}_{xx}+\sum_{n=0}^{\infty }p^{n}H_{n}\left(\theta ,\eta \right)\right]\right]\text{.}$$

on comparing powers of p in Equation (2.41):

$$p^{0}:\theta_{0}\left(x,t\right)=e^{x}\text{.}$$

(2.42) $$p^{1}:\theta_{1}\left(x,t\right)=S^{-1}\left[\theta S\left\{{\left(\theta_{0}\right)}_{xx}+H_{0}\left(\theta ,\eta \right)\right\}\right]\text{.}$$

where, $H_{0}\left(\theta ,\eta \right)={\left(\theta_{0}\right)}_{x}\;\eta_{0}+\theta_{0}{\left(\eta_{0}\right)}_{x}-2\;\theta_{0}{\left(\theta_{0}\right)}_{x}=0\text{.}$.

By using the value of $H_{0}\left(\theta ,\eta \right)=0$ in Equation (2.42):

$$\theta_{1}\left(x,t\right)=S^{-1}\left[\theta S\left\{{\left(\theta_{0}\right)}_{xx}\right\}\right]\Rightarrow \theta_{1}\left(x,t\right)=S^{-1}\left[\theta S\left\{e^{x}\right\}\right]\text{,}$$

$$\Rightarrow \theta_{1}\left(x,t\right)=S^{-1}\left[e^{x}\;\theta \right]\Rightarrow \theta_{1}\left(x,t\right)=e^{x}\;S^{-1}\left[\theta \right]\text{,}$$

$$\Rightarrow \theta_{1}\left(x,t\right)=e^{x}\;t\text{.}$$

(2.43) $$p^{2}:{\theta_{2}\left(x,t\right)=S}^{-1}\left[\theta S\left\{{\left(\theta_{1}\right)}_{xx}+H_{1}\left(\theta ,\eta \right)\right\}\right]\text{.}$$

where,

$$H_{1}\left(\theta ,\eta \right)={\left(\theta_{1}\right)}_{x}\;\eta_{0}+{\left(\theta_{0}\right)}_{x}\eta_{1}+\left(\theta_{1}\right){\left(\eta_{0}\right)}_{x}+\theta_{0}\;{\left(\eta_{1}\right)}_{x}-2\;\left[\theta_{1}{\left(\theta_{0}\right)}_{x}+\theta_{0}{\left(\theta_{1}\right)}_{x}\right]=0$$

By using the value of $H_{1}\left(\theta ,V\right)=0$ in Equation (2.43):

$${\theta_{2}\left(x,t\right)=S}^{-1}\left[\theta S\left\{{\left(\theta_{1}\right)}_{xx}\right\}\right]\Rightarrow {\theta_{2}\left(x,t\right)=S}^{-1}\left[\theta S\left\{e^{x}t\right\}\right]$$

$$\Rightarrow {\theta_{2}\left(x,t\right)=e^{x}\;S}^{-1}\left[\theta S\left\{t\right\}\right]\Rightarrow {\theta_{2}\left(x,t\right)=e^{x}\;S}^{-1}\left[\theta^{2}\right]\text{,}$$

$$\Rightarrow \theta_{2}\left(x,t\right)=\frac{e^{x}t^{2}}{2}\text{.}$$

in general, $\theta \left(x,t\right)=\theta_{0}\left(x,t\right)+\theta_{1}\left(x,t\right)+\theta_{2}\left(x,t\right)+\ldots$.

$$\theta \left(x,t\right)=e^{x}+e^{x}t+\frac{e^{x}t^{2}}{2}+\ldots$$

(2.44) $$\theta \left(x,t\right)=e^{x+t}\text{.}$$

in Equation (2.44), exact solution is notified.

On applying Sumudu transform on Equation (2.39):

$$S[\eta_{t}]={S[\eta }_{xx}-2\;\eta \;\eta_{x}+{\left[\theta \eta \right]}_{x}]\text{,}$$

$$\frac{1}{\eta }\left[S\left(\eta \left(x,t\right)\right)-\eta \left(x,0\right)\right]={S[\eta }_{xx}-2\;\eta \;\eta_{x}+{\left[\theta \eta \right]}_{x}]\text{,}$$

$$\left[S\left(\eta \left(x,t\right)\right)-\eta \left(x,0\right)\right]=\eta [{S[\eta }_{xx}-2\;\eta \;\eta_{x}+{\left[\theta \eta \right]}_{x}]]\text{,}$$

$$\left[S\left(\eta \left(x,t\right)\right)\right]=\eta \left(x,0\right)+\eta \left[{S[\eta }_{xx}-2\;\eta \;\eta_{x}+{\left[\theta \eta \right]}_{x}\right]\text{,}$$

$$\left[S\left(\eta \left(x,t\right)\right)\right]=e^{x}+\eta \left[{S[\eta }_{xx}-2\;\eta \;\eta_{x}+{\left[\theta \eta \right]}_{x}\right]\text{,}$$

$$\eta \left(x,t\right)=e^{x}+S^{-1}\left[\eta \left[{S[\eta }_{xx}-2\;\eta \;\eta_{x}+{\left[\theta \eta \right]}_{x}\right]\right]\text{,}$$

(2.45) $$\eta \left(x,t\right)=e^{x}+S^{-1}\left[\eta \left[S\left[\eta_{xx}+I_{n}\left(\theta ,\eta \right)\right]\right]\right]\text{.}$$

where $I_{n}\left(\theta ,\eta \right)={\left(\theta \eta \right)}_{x}-2\;\eta \;\eta_{x}$ , Where $I_{n}\left(\theta ,\eta \right)$ is representing non-linear term.

$$\Rightarrow I_{n}\left(\theta ,\eta \right)=\left(\theta_{x}\eta +\theta \;\eta_{x}\right)-2\;\eta \;\eta_{x}\text{.}$$

By applying HPM in Equation (2.45):

(2.46) $$\Rightarrow \sum_{n=0}^{\infty }p^{n}\eta_{n}\left(x,t\right)=e^{x}+pS^{-1}\left[\eta S\left[{\left\{\sum_{n=0}^{\infty }p^{n}\eta_{n}\left(x,t\right)\right\}}_{xx}+\sum_{n=0}^{\infty }p^{n}I_{n}\left(\theta ,\eta \right)\right]\right]\text{.}$$

On comparing powers of p in Equation (2.46):

$$p^{0}:\eta_{0}\left(x,t\right)=e^{x}\text{.}$$

(2.47) $$p^{1}:\eta_{1}\left(x,t\right)=S^{-1}\left[\eta S\left\{{\left(\eta_{0}\right)}_{xx}+I_{0}\left(\theta ,\eta \right)\right\}\right]\text{.}$$

where, $I_{0}\left(\theta ,\eta \right)={\left(\theta_{0}\right)}_{x}\;\eta_{0}+\theta_{0}{\left(\eta_{0}\right)}_{x}-2\;\eta_{0}{\left(\eta_{0}\right)}_{x}=0\text{.}$.

By using the value of $I_{0}\left(\theta ,\eta \right)=0$ in Equation (2.47):

$$\eta_{1}\left(x,t\right)=S^{-1}\left[\eta S\left\{{\left(\eta_{0}\right)}_{xx}\right\}\right]\Rightarrow \eta_{1}\left(x,t\right)=S^{-1}\left[\eta S\left\{e^{x}\right\}\right]\text{,}$$

$$\Rightarrow \eta_{1}\left(x,t\right)=S^{-1}\left[e^{x}\;\eta \right]\Rightarrow \eta_{1}\left(x,t\right)=e^{x}\;S^{-1}\left[\eta \right]\text{,}$$

(2.48) $$\eta_{1}\left(x,t\right)=e^{x}\;t\text{.}$$

Via Equation (2.48), the second term of the series approximation is provided.

(2.49) $$p^{2}:{\eta_{2}\left(x,t\right)=S}^{-1}\left[\eta S\left\{{\left(\eta_{1}\right)}_{xx}+I_{1}\left(\theta ,\eta \right)\right\}\right]\text{.}$$

where, $I_{1}\left(\theta ,\eta \right)={\left(\theta_{1}\right)}_{x}\;\eta_{0}+{\left(\theta_{0}\right)}_{x}\eta_{1}+\left(\theta_{1}\right){\left(\eta_{0}\right)}_{x}+\theta_{0}\;{\left(\eta_{1}\right)}_{x}-2\;\left[\eta_{1}{\left(\eta_{0}\right)}_{x}+\eta_{0}{\left(\eta_{1}\right)}_{x}\right]=0\text{.}$.

Using $I_{1}\left(\theta ,\eta \right)=0$ in Equation (2.49):

$${\eta_{2}\left(x,t\right)=S}^{-1}\left[\eta S\left\{{\left(\eta_{1}\right)}_{xx}\right\}\right]\Rightarrow {\eta_{2}\left(x,t\right)=S}^{-1}\left[\eta S\left\{{\left(\eta_{1}\right)}_{xx}\right\}\right]\text{,}$$

$$\Rightarrow {\eta_{2}\left(x,t\right)=S}^{-1}\left[\eta S\left\{e^{x}t\right\}\right]\Rightarrow {\eta_{2}\left(x,t\right)=e^{x}\;S}^{-1}\left[\eta S\left\{t\right\}\right]\text{,}$$

$$\Rightarrow {\eta_{2}\left(x,t\right)=e^{x}\;S}^{-1}\left[\eta^{2}\right]\text{,}$$

$$\eta_{2}\left(x,t\right)=\frac{e^{x}t^{2}}{2}\text{.}$$

in general, $\eta \left(x,t\right)=\eta_{0}\left(x,t\right)+\eta_{1}\left(x,t\right)+\eta_{2}\left(x,t\right)+\ldots$.

$$\eta \left(x,t\right)=e^{x}+e^{x}t+\frac{e^{x}t^{2}}{2}+\ldots$$

$$\eta \left(x,t\right)=e^{x+t}\text{.}$$

Exact solutions in this example are provided as follows via Equation (2.50)–(2.51):

(2.50) $$\theta \left(x,t\right)=e^{x+t}\text{,}$$

(2.51) $$\eta \left(x,t\right)=e^{x+t}\text{.}$$

• Application of Elzaki HPM [Method II]

Applying Elzaki transform on Equation (2.38),

$$E\left[\theta_{t}\right]=E\left[\theta_{xx}+{\left(\theta \eta \right)}_{x}-2\;\theta \;\theta_{x}\right]\text{,}$$

$$\Rightarrow \frac{1}{\nu }\left[E\left\{\theta \left(x,t\right)\right\}\right]-\nu \;\theta \left(x,0\right)=E\;\left[\theta_{xx}+{\left(\theta \eta \right)}_{x}-2\theta \;\theta_{x}\right]\text{,}$$

$$\Rightarrow E\left[\theta \left(x,t\right)\right]=\nu^{2}\;\theta \left(x,0\right)+\nu E\left[\theta_{xx}+{\left(\theta \eta \right)}_{x}-2\;\theta \;\theta_{x}\right]\text{,}$$

$$\Rightarrow \theta \left(x,t\right)=\theta \left(x,0\right)E^{-1}\left[\nu^{2}\right]+E^{-1}\left[\nu E\left\{\theta_{xx}+{\left(\theta \eta \right)}_{x}-2\;\theta \theta_{x}\right\}\right]\text{,}$$

(2.52) $$\Rightarrow \theta \left(x,t\right)=e^{x}+E^{-1}\left[\nu E\left\{\theta_{xx}+{\left(\theta \eta \right)}_{x}-2\;\theta \;\theta_{x}\right\}\right]\text{.}$$

Applying HPM in Equation (2.52)

$$\sum_{n=0}^{\infty }\theta_{n}\left(x,t\right)=e^{x}+p\;E^{-1}\left[\nu E\left\{{\left(\sum_{n=0}^{\infty }p^{n}\theta_{n}\left(x,t\right)\right)}_{xx}+\sum_{n=0}^{\infty }H_{n}\left(\theta ,\eta \right)\right\}\right]\text{.}$$

where $H_{n}\left(\theta ,\eta \right)$ is considered as the non-linear term.

$$H_{n}\left(\theta ,\eta \right)={\left(\theta \eta \right)}_{x}-2\;\theta \;\theta_{x}\text{,}$$

(2.53) $$H_{0}\left(\theta ,\eta \right)={\left(\theta_{0}\right)}_{x}\eta_{0}+\theta_{0}{\left(\eta_{0}\right)}_{x}-2\;\theta_{0}{\left(\theta_{0}\right)}_{x}\text{.}$$

Comparing powers of p in Equation (2.53),

$$p^{0}:\theta_{0}\left(x,t\right)=e^{x}\text{.}$$

$$p^{1}:\theta_{1}\left(x,t\right)=E^{-1}\left[\nu E\left\{{\left(\theta_{0}\left(x,t\right)\right)}_{xx}+H_{0}\left(\theta ,\eta \right)\right\}\right]\text{,}$$

$$\Rightarrow \theta_{1}\left(x,t\right)=E^{-1}\left[\nu E\left\{e^{x}\right\}\right]\Rightarrow \theta_{1}\left(x,t\right)=E^{-1}\left[\nu E\left(e^{x}\right)\right]\text{,}$$

$$\Rightarrow \theta_{1}\left(x,t\right)=E^{-1}\left[e^{x}\nu E\left(1\right)\right]\Rightarrow \theta_{1}\left(x,t\right)=e^{x}E^{-1}\left[\nu^{3}\right]\text{,}$$

$$\Rightarrow \theta_{1}\left(x,t\right)=te^{x}\text{.}$$

$$p^{2}:\theta_{2}\left(x,t\right)=E^{-1}\left[\nu E\left\{{\left(\theta_{1}\left(x,t\right)\right)}_{xx}+H_{1}\left(\theta ,\eta \right)\right\}\right]\text{,}$$

(2.54) $$\Rightarrow \theta_{2}\left(x,t\right)=E^{-1}\left[\nu E\left\{t\;e^{x}\right\}\right]\text{,}$$

where, $H_{1}=\left[{\left(\theta_{1}\right)}_{x}\eta_{0}+{\left(\theta_{0}\right)}_{x}\eta_{1}\right]+\left[\theta_{1}{\left(\eta_{0}\right)}_{x}+\theta_{0}{\left(\eta_{1}\right)}_{x}\right]-2\;\left[\theta_{1}{\left(\theta_{0}\right)}_{x}+\theta_{0}{\left(\theta_{1}\right)}_{x}\right]=0\text{.}$.

From Equation (2.54),

$$\Rightarrow \theta_{2}\left(x,t\right)=e^{x}E^{-1}\left[\nu E\left\{t\right\}\right]\Rightarrow \theta_{2}\left(x,t\right)=e^{x}E^{-1}\left[\nu^{4}\right]\text{,}$$

$$\Rightarrow \theta_{2}\left(x,t\right)=\frac{t^{2}}{∠2}e^{x}\text{.}$$

$$\theta \left(x,t\right)=\theta_{0}\left(x,t\right)+\theta_{1}\left(x,t\right)+\theta_{2}\left(x,t\right)+\ldots$$

$$\theta \left(x,t\right)=e^{x}+te^{x}+\frac{t^{2}}{∠2}e^{x}+\ldots$$

$$\theta \left(x,t\right)=e^{x+t}\text{.}$$

similarly, Applying Elzaki transform on Equation (2.39),

$$E\left[\eta_{t}\right]=E\left[\eta_{xx}+{\left(\theta \eta \right)}_{x}-2\;\eta \;\eta_{x}\right]\text{,}$$

$$\Rightarrow \frac{1}{\nu }\left[E\;\eta \left(x,t\right)\right]-\nu \;\eta \left(x,0\right)=E\;\left[\eta_{xx}+{\left(\theta \eta \right)}_{x}-2\eta \;V_{x}\right]\text{,}$$

$$\Rightarrow E\left[\eta \left(x,t\right)\right]=\nu^{2}\;\eta \left(x,0\right)+\nu E\left[\eta_{xx}+{\left(\theta \eta \right)}_{x}-2\;\eta \;\eta_{x}\right]\text{,}$$

$$\Rightarrow \eta \left(x,t\right)=\eta \left(x,0\right)E^{-1}\left[\nu^{2}\right]+E^{-1}\left[\nu E\left\{\eta_{xx}+{\left(\theta \eta \right)}_{x}-2\;\eta \eta_{x}\right\}\right]\text{,}$$

(2.55) $$\Rightarrow \eta \left(x,t\right)=e^{x}+E^{-1}\left[\nu E\left\{\eta_{xx}+{\left(\theta \eta \right)}_{x}-2\;\eta \;\eta_{x}\right\}\right]\text{.}$$

applying HPM in Equation (2.55),

$$\sum_{n=0}^{\infty }\eta_{n}\left(x,t\right)=e^{x}+p\;E^{-1}\left[\nu E\left\{{\left(\sum_{n=0}^{\infty }p^{n}\eta_{n}\left(x,yt\right)\right)}_{xx}+\sum_{n=0}^{\infty }I_{n}\left(\theta ,\eta \right)\right\}\right]\text{.}$$

where $I_{n}\left(U,\eta \right)$ is considered as the non-linear term.

$$I_{n}\left(\theta ,\eta \right)={\left(\theta \eta \right)}_{x}-2\;\eta \;\eta_{x}\text{,}$$

(2.56) $$I_{0}\left(\theta ,\eta \right)={\left(\theta_{0}\right)}_{x}\eta_{0}+\theta_{0}{\left(\eta_{0}\right)}_{x}-2\;\eta_{0}{\left(\eta_{0}\right)}_{x}\text{.}$$

Comparing powers of p in Equation (2.56),

$$p^{0}:\eta_{0}\left(x,t\right)=e^{x}\text{.}$$

$$p^{1}:\eta_{1}\left(x,t\right)=E^{-1}\left[\nu E\left\{{\left(\eta_{0}\left(x,t\right)\right)}_{xx}+I_{0}\left(\theta ,\eta \right)\right\}\right]\text{,}$$

$$\Rightarrow \eta_{1}\left(x,t\right)=E^{-1}\left[\nu E\left\{e^{x}\right\}\right]\Rightarrow \eta_{1}\left(x,t\right)=E^{-1}\left[\nu E\left(e^{x}\right)\right]\text{,}$$

$$\Rightarrow \eta_{1}\left(x,t\right)=E^{-1}\left[e^{x}\nu E\left(1\right)\right]\Rightarrow \eta_{1}\left(x,t\right)=e^{x}E^{-1}\left[\nu^{3}\right]\text{,}$$

$$\Rightarrow \eta_{1}\left(x,t\right)=te^{x}\text{.}$$

$$p^{2}:\eta_{2}\left(x,t\right)=E^{-1}\left[\nu E\left\{{\left(\eta_{1}\left(x,t\right)\right)}_{xx}+I_{1}\left(\theta ,\eta \right)\right\}\right]\text{,}$$

(2.57) $$\Rightarrow \eta_{2}\left(x,t\right)=E^{-1}\left[\nu E\left\{t\;e^{x}\right\}\right]\text{.}$$

where, $I_{1}=\left[{\left(\theta_{1}\right)}_{x}\eta_{0}+{\left(\theta_{0}\right)}_{x}\eta_{1}\right]+\left[\theta_{1}{\left(\eta_{0}\right)}_{x}+\theta_{0}{\left(\eta_{1}\right)}_{x}\right]-2\;\left[\eta_{1}{\left(\eta_{0}\right)}_{x}+\eta_{0}{\left(\eta_{1}\right)}_{x}\right]=0\text{.}$.

From Equation (2.57),

$$\Rightarrow \eta_{2}\left(x,t\right)=e^{x}E^{-1}\left[\nu E\left\{t\right\}\right]\Rightarrow \eta_{2}\left(x,t\right)=e^{x}E^{-1}\left[\nu^{4}\right]\text{,}$$

$$\Rightarrow \eta_{2}\left(x,t\right)=\frac{t^{2}}{∠2}e^{x}\text{.}$$

$$\eta \left(x,t\right)=\eta_{0}\left(x,t\right)+\eta_{1}\left(x,t\right)+\eta_{2}\left(x,t\right)+\ldots$$

$$\eta \left(x,t\right)=e^{x}+te^{x}+\frac{t^{2}}{∠2}e^{x}+\ldots$$

$$\eta \left(x,t\right)=e^{x+t}\text{.}$$

Exact solutions are as follows:

$$\theta \left(x,t\right)=e^{x+t}\;and\;\eta \left(x,t\right)=e^{x+t}\text{.}$$

### 3.2 Convergence analysis

$\mu_{0}=\frac{\left\|\theta_{1}\right\|}{\left\|\theta_{0}\right\|}$ = 1.0000E-02 < 1, $\mu_{1}=\frac{\left\|\theta_{2}\right\|}{\left\|\theta_{1}\right\|}$ = 5.0000E-03 < 1

$\mu_{2}=\frac{\left\|\theta_{3}\right\|}{\left\|\theta_{2}\right\|}$ = 3.3333E-03 < 1, $\mu_{3}=\frac{\left\|\theta_{4}\right\|}{\left\|\theta_{3}\right\|}$ = 2.5000E-03 < 1

and $\nu_{0}=\frac{\left\|\eta_{1}\right\|}{\left\|\eta_{0}\right\|}$ = 1.0000E-02 < 1, $\nu_{1}=\frac{\left\|\eta_{2}\right\|}{\left\|\eta_{1}\right\|}$ = 5.0000E-03 < 1

$\nu_{2}=\frac{\left\|\eta_{3}\right\|}{\left\|\eta_{2}\right\|}$ = 3.3333E-03 < 1, $\nu_{3}=\frac{\left\|\eta_{4}\right\|}{\left\|\eta_{3}\right\|}$ = 2.5000E-03 < 1

$\mu_{i}^{\prime }s$ and ${\nu_{i}}^{\prime }s$ for i≥0 and $t\leq \left(\frac{j}{2}\right)$ for 0<j<1 are less than one. Hence convergence condition is completed.

Example 3.

In this example, coupled 2D Burgers’ equation is considered as follows [68]:

$$\theta_{t}+\theta \;\theta_{x}+\eta \;\theta_{y}=\frac{1}{Re}\left[\theta_{xx}+\theta_{yy}\right]\text{,}$$

$$\eta_{t}+\theta \;\eta_{x}+\eta \;\eta_{y}=\frac{1}{Re}\left[\eta_{xx}+\eta_{yy}\right]\text{.}$$

(2.58) $$or\;\theta_{t}=\frac{1}{Re}\left[\theta_{xx}+\theta_{yy}\right]-\theta \;\theta_{x}-\eta \;\theta_{y}\text{,}$$

(2.59) $$\eta_{t}=\frac{1}{Re}\left[\eta_{xx}+\eta_{yy}\right]-\theta \;\eta_{x}-\eta \;\eta_{y}\text{.}$$

(2.60) I.C.s:θ(x,y,0)=−sin(x+y),

(2.61) η(x,y,0)=sin(x+y).

Equations (2.60)–(2.61) are provided as initial conditions.

• Application of Sumudu HPM [Method I]

Applying Sumudu transform in Equation (2.58):

$$S\left[\theta_{t}\right]=S\left[\frac{1}{Re}\left[\theta_{xx}+\theta_{yy}\right]-\theta \;\theta_{x}-\eta \;\theta_{y}\right]\text{,}$$

$$\Rightarrow \frac{1}{\theta }\left[S(\theta \left(x,y,t\right)-\theta \left(x,y,0\right)\right]=S\left[\frac{1}{Re}\left[\theta_{xx}+\theta_{yy}\right]-\left(\theta \;\theta_{x}+\eta \;\theta_{y}\right)\right]\text{,}$$

$$\Rightarrow S(\theta \left(x,y,t\right)-\theta \left(x,y,0\right)=\theta \left[S\left[\frac{1}{Re}\left[\theta_{xx}+\theta_{yy}\right]-\left(\theta \;\theta_{x}+\eta \;\theta_{y}\right)\right]\right]\text{,}$$

$$\Rightarrow S\left(\theta \left(x,y,t\right)\right)=\theta \left(x,y,0\right)+\theta \left[S\left[\frac{1}{Re}\left[\theta_{xx}+\theta_{yy}\right]-\left(\theta \;\theta_{x}+\eta \;\theta_{y}\right)\right]\right]$$

(2.62) $$\theta \left(x,y,t\right)=\theta \left(x,y,0\right)+S^{-1}\left[\theta \left[S\left[\frac{1}{Re}\left[\theta_{xx}+\theta_{yy}\right]-\left(\theta \;\theta_{x}+\eta \;\theta_{y}\right)\right]\right]\right]\text{.}$$

Applying HPM in Equation (2.62):

(2.63) $$\sum_{n=0}^{\infty }p^{n}\theta_{n}\left(x,y,t\right)=-\sin\left(x+y\right)+p\;S^{-1}\left[\theta S\left\{\frac{1}{Re}\left({\left(\sum_{n=0}^{\infty }p^{n}\theta_{n}\right)}_{xx}+{\left(\sum_{n=0}^{\infty }p^{n}\theta_{n}\right)}_{yy}\right)-\sum_{n=0}^{\infty }p^{n}H_{n}\left(\theta ,\eta \right)\right\}\right]\text{.}$$

Where, $H_{n}\left(\theta ,\eta \right)=\theta \;\theta_{x}+\eta \;\theta_{y}$ , $H_{n}\left(\theta ,\eta \right)$ is a non-linear term.

$$H_{0}\left(\theta ,\eta \right)=\theta_{0}\;{\left(\theta_{0}\right)}_{x}+\eta_{0}\;{\left(\theta_{0}\right)}_{y}\text{.}$$

On comparing powers of p in Equation (2.63):

$$p^{0}:\theta_{0}\left(x,y,t\right)=-\sin\left(x+y\right)\text{.}$$

$$p^{1}:\theta_{1}\left(x,y,t\right)=S^{-1}\left[\theta S\{\frac{1}{Re}(({\theta_{0}}_{)}+\left({\theta_{0}}_{)}\right)-H_{0}\left(\theta ,\eta \right)\}\right]\text{,}$$

$$\Rightarrow \theta_{1}\left(x,y,t\right)=S^{-1}\left[\theta S\left\{\frac{1}{Re}\left(2\;\sin\left(x+y\right)\right)\right\}\right]\text{,}$$

$$\Rightarrow \theta_{1}\left(x,y,t\right)=\frac{2}{Re}\sin\left(x+y\right)S^{-1}\left[\theta S\left(1\right)\right]\text{,}$$

$$\Rightarrow \theta_{1}\left(x,y,t\right)=\frac{2}{Re}\sin\left(x+y\right)S^{-1}\left[\theta \right]\text{,}$$

$$\Rightarrow \theta_{1}\left(x,y,t\right)=\frac{2t}{Re}\sin\left(x+y\right)\text{.}$$

$$p^{2}:\theta_{2}\left(x,y,t\right)=S^{-1}\left[\theta S\{\frac{1}{Re}(({\theta_{1}}_{)}+\left({\theta_{1}}_{)}\right)-H_{1}\left(\theta ,\eta \right)\}\right]\text{.}$$

Where, $H_{1}\left(\theta ,\eta \right)=\left[\theta_{1}{\left(\theta_{0}\right)}_{x}+\theta_{0}{\left(\theta_{1}\right)}_{x}\right]+\left[\eta_{1}{\left(\theta_{0}\right)}_{y}+\eta_{0}{\left(\theta_{1}\right)}_{y}\right]=0\text{.}$.

$$\theta_{2}\left(x,y,t\right)=S^{-1}\left[\theta S\left\{\frac{1}{Re}\left(-\frac{4t}{Re}\sin\left(x+y\right)\right)\right\}\right]\text{,}$$

$$\Rightarrow \theta_{2}\left(x,y,t\right)=-\frac{4}{Re^{2}}\sin\left(x+y\right)S^{-1}\left[\theta S\left(t\right)\right]\text{,}$$

$$\Rightarrow \theta_{2}\left(x,y,t\right)=-\frac{4}{Re^{2}}\sin\left(x+y\right)S^{-1}\left[\theta^{2}\right]\text{,}$$

$$\Rightarrow \theta_{2}\left(x,y,t\right)=-\frac{4}{Re^{2}}\sin\left(x+y\right)\;\left(\frac{t^{2}}{∠2}\right)\text{.}$$

In general, $\theta \left(x,y\;t\right)=\theta_{0}\left(x,y,t\right)+\theta_{1}\left(x,y,t\right)+\theta_{2}\left(x,y,t\right)+\ldots$.

$$\theta \left(x,y,t\right)=-\sin\left(x+y\right)+\frac{2t}{Re}\sin\left(x+y\right)-\frac{4}{Re^{2}}\sin\left(x+y\right)\;\left(\frac{t^{2}}{∠2}\right)+\ldots$$

θ(x,y,t)=−sin(x+y)exp(−2νt).

Similarly, applying Sumudu transform on Equation (2.59):

$$S\left[\eta_{t}\right]=S\left[\frac{1}{Re}\left[\eta_{xx}+\eta_{yy}\right]-\theta \;\eta_{x}-\eta \;\eta_{y}\right]\text{,}$$

$$\Rightarrow \frac{1}{\eta }\left[S(\eta \left(x,y,t\right)-\eta \left(x,y,0\right)\right]=S\left[\frac{1}{Re}\left[\eta_{xx}+\eta_{yy}\right]-\left(\theta \;\eta_{x}+\eta \;\eta_{y}\right)\right]\text{,}$$

$$\Rightarrow S(\eta \left(x,y,t\right)-\eta \left(x,y,0\right)=\eta \left[S\left[\frac{1}{Re}\left[\eta_{xx}+\eta_{yy}\right]-\left(\theta \;\eta_{x}+\eta \;\eta_{y}\right)\right]\right]\text{,}$$

$$\Rightarrow S\left(\eta \left(x,y,t\right)\right)=\eta \left(x,y,0\right)+\eta \left[S\left[\frac{1}{Re}\left[\eta_{xx}+\eta_{yy}\right]-\left(\theta \;\eta_{x}+\eta \;\eta_{y}\right)\right]\right]\text{,}$$

$$\Rightarrow \eta \left(x,y,t\right)=\eta \left(x,y,0\right)+S^{-1}\left[\eta \left[S\left[\frac{1}{Re}\left[\eta_{xx}+\eta_{yy}\right]-\left(\theta \;\eta_{x}+\eta \;\eta_{y}\right)\right]\right]\right]\text{,}$$

(2.64) $$\eta \left(x,y,t\right)=\sin\left(x+y\right)+S^{-1}\left[\eta \left[S\left[\frac{1}{Re}\left[\eta_{xx}+\eta_{yy}\right]-\left(\theta \;\eta_{x}+\eta \;\eta_{y}\right)\right]\right]\right]\text{.}$$

Applying HPM in Equation (2.64):

(2.65) $$\sum_{n=0}^{\infty }p^{n}\eta_{n}\left(x,y,t\right)=\sin\left(x+y\right)+p\;S^{-1}\left[\eta S\left\{\frac{1}{Re}\left({\left(\sum_{n=0}^{\infty }p^{n}\eta_{n}\right)}_{xx}+{\left(\sum_{n=0}^{\infty }p^{n}\eta_{n}\right)}_{yy}\right)-\sum_{n=0}^{\infty }p^{n}I_{n}\left(\theta ,\eta \right)\right\}\right]\text{.}$$

Where, $I_{n}\left(\theta ,\eta \right)=\theta \;\eta_{x}+\eta \;\eta_{x}$, Where $I_{n}\left(\theta ,\eta \right)$ is a non-linear term.

$$I_{0}\left(\theta ,\eta \right)=\theta_{0}\;{\left(\eta_{0}\right)}_{x}+\eta_{0}\;{\left(\eta_{0}\right)}_{x}=0\text{.}$$

Comparing powers of p in Equation (2.65):

$$p^{0}:\eta_{0}\left(x,y,t\right)=\sin\left(x+y\right)\text{.}$$

$$p^{1}:\eta_{1}\left(x,y,t\right)=S^{-1}\left[\eta S\{\frac{1}{Re}(({\eta_{0}}_{)}+\left({\eta_{0}}_{)}\right)-I_{0}\left(\theta ,\eta \right)\}\right]\text{,}$$

$$\Rightarrow \eta_{1}\left(x,y,t\right)=S^{-1}\left[\eta S\left\{\frac{1}{Re}\left(-2\;\sin\left(x+y\right)\right)\right\}\right]\text{,}$$

$$\Rightarrow \eta_{1}\left(x,y,t\right)=\frac{-2}{Re}\sin\left(x+y\right)S^{-1}\left[\eta S\left(1\right)\right]\text{,}$$

$$\Rightarrow \eta_{1}\left(x,y,t\right)=\frac{-2}{Re}\sin\left(x+y\right)S^{-1}\left[\eta \right]\text{,}$$

$$\Rightarrow \eta_{1}\left(x,y,t\right)=\frac{-2t}{Re}\sin\left(x+y\right)\text{.}$$

$$p^{2}:\eta_{2}\left(x,y,t\right)=S^{-1}\left[\eta S\{\frac{1}{Re}(({\eta_{1}}_{)}+\left({\eta_{1}}_{)}\right)-I_{1}\left(\theta ,\eta \right)\}\right]\text{,}$$

$$\eta_{2}\left(x,y,t\right)=S^{-1}\left[\eta S\left\{\frac{1}{Re}\left(\frac{4t}{Re}\sin\left(x+y\right)\right)\right\}\right]\text{,}$$

$$\Rightarrow \eta_{2}\left(x,y,t\right)=\frac{4}{Re^{2}}\sin\left(x+y\right)S^{-1}\left[\eta S\left(t\right)\right]\text{,}$$

$$\Rightarrow \eta_{2}\left(x,y,t\right)=\frac{4}{Re^{2}}\sin\left(x+y\right)S^{-1}\left[\eta^{2}\right]\text{,}$$

$$\Rightarrow \eta_{2}\left(x,y,t\right)=\frac{4}{Re^{2}}\sin\left(x+y\right)\;\left(\frac{t^{2}}{∠2}\right)\text{.}$$

$$\eta \left(x,y\;t\right)=\eta_{0}\left(x,y,t\right)+\eta_{1}\left(x,y,t\right)+\eta_{2}\left(x,y,t\right)+\ldots$$

$$\eta \left(x,y,t\right)=\sin\left(x+y\right)-\frac{2t}{Re}\sin\left(x+y\right)+\frac{4}{Re^{2}}\sin\left(x+y\right)\;\left(\frac{t^{2}}{∠2}\right)-\ldots$$

η(x,y,t)=sin(x+y)exp(−2νt).

Exact solutions are:

(2.66) θ(x,y,t)=−sin(x+y)exp(−2νt),

(2.67) η(x,y,t)=sin(x+y)exp(−2νt).

Via Equations (2.66)–(2.67), exact solutions are provided.

• Application of Elzaki HPM [Method II]

Applying Elzaki transform upon equation (2.58)

$$E\left[\theta_{t}\right]=E\left[\frac{1}{Re}\left\{\theta_{xx}+\theta_{yy}\right\}-\theta \;\theta_{x}-\eta \;\theta_{y}\right]\text{,}$$

$$\Rightarrow \frac{1}{\nu }E\left[\theta \left(x,y,t\right)\right]-\nu \;\theta \left(x,y,0\right)=E\left[\frac{1}{Re}\left\{\theta_{xx}+\theta_{yy}\right\}-\theta \;\theta_{x}-\eta \;\theta_{y}\right]\text{,}$$

$$\Rightarrow E\left[\theta \left(x,y,t\right)\right]=\nu^{2}\theta \left(x,y,0\right)+\nu E\left[\frac{1}{Re}\left\{\theta_{xx}+\theta_{yy}\right\}-\theta \;\theta_{x}-\eta \;\theta_{y}\right]\text{,}$$

$$\Rightarrow \theta \left(x,y,t\right)=\theta \left(x,y,0\right)E^{-1}\left(\nu^{2}\right)+E^{-1}\left[\nu E\left[\frac{1}{Re}\left\{\theta_{xx}+\theta_{yy}\right\}-\theta \;\theta_{x}-\eta \;\theta_{y}\right]\right]\text{,}$$

(2.68) $$\theta \left(x,y,t\right)=-\sin\left(x+y\right)+E^{-1}\left[\nu E\left[\frac{1}{Re}\left\{\theta_{xx}+\theta_{yy}\right\}-\theta \;\theta_{x}-\eta \;\theta_{y}\right]\right]\text{.}$$

Applying HPM in Equation (2.68),

(2.69) $$\sum_{n=0}^{\infty }p^{n}\theta_{n}\left(x,y,t\right)=-\sin\left(x+y\right)+p\;E^{-1}\left[\nu E\{\frac{1}{Re}\left\{{\left(\sum_{n=0}^{\infty }\theta_{n}\left(x,t\right)\right)}_{xx}+{\left(\sum_{n=0}^{\infty }\theta_{n}\left(x,t\right)\right)}_{yy}\right\}-H_{n}\left(\theta ,\eta \right)\right]\text{.}$$

where $H_{n}\left(\theta ,\eta \right)$ is the non-linear term.

$$H_{n}\left(\theta ,\eta \right)=\theta \;\theta_{x}+\eta \;\theta_{y}\text{.}$$

Comparing powers of p in Equation (2.69),

$$p^{0}:\theta_{0}\left(x,y,t\right)=-\sin\left(x+y\right)\text{.}$$

(2.70) $$p^{1}:\theta_{1}\left(x,y,t\right)=E^{-1}\left[\nu E\left\{\frac{1}{Re}\left\{{\left(\theta_{0}\left(x,t\right)\right)}_{xx}+{\left(\theta_{0}\left(x,t\right)\right)}_{yy}\right\}-H_{0}\left(\theta ,\eta \right)\right\}\right]\text{.}$$

where, $H_{0}\left(\theta ,\eta \right)=\theta_{0}{\left(\theta_{0}\right)}_{x}+\eta_{0}{\left(\theta_{0}\right)}_{y}$ = 0.

From Equation (2.70),

$$\theta_{1}\left(x,y,t\right){=E}^{-1}\left[\nu E\left\{\frac{1}{Re}\left(2\;\sin\left(x+y\right)\right)\right\}\right]\text{,}$$

$$\Rightarrow \theta_{1}\left(x,y,t\right){=\frac{2}{Re}\sin\left(x+y\right)E}^{-1}\left[\nu E\left\{1\right\}\right]\text{,}$$

$$\Rightarrow \theta_{1}\left(x,y,t\right){=\frac{2}{Re}\sin\left(x+y\right)E}^{-1}\left[\nu^{3}\}\right]\text{,}$$

$$\Rightarrow \theta_{1}\left(x,y,t\right)=\frac{2t}{Re}\sin\left(x+y\right)\text{.}$$

(2.71) $$p^{2}:\theta_{2}\left(x,y,t\right)=E^{-1}\left[\nu E\left\{\frac{1}{Re}\left\{{\left(\theta_{1}\left(x,t\right)\right)}_{xx}+{\left(\theta_{1}\left(x,t\right)\right)}_{yy}\right\}-H_{1}\left(\theta ,\eta \right)\right\}\right]\text{.}$$

where, $H_{1}\left(\theta ,\eta \right)=\left[\theta_{1}{\left(\theta_{0}\right)}_{x}+\theta_{0}{\left(\theta_{1}\right)}_{x}\right]+\left[\eta_{1}{\left(\theta_{0}\right)}_{y}+\eta_{0}{\left(\theta_{1}\right)}_{y}\right]=0\text{.}$.

From Equation (2.71),

$$\theta_{2}\left(x,y,t\right)=E^{-1}\left[\nu E\left\{-\frac{4t}{Re^{2}}\sin\left(x+y\right)\right\}\right]\text{,}$$

$$\Rightarrow \theta_{2}\left(x,y,t\right)=-\frac{4}{Re^{2}}\sin\left(x+y\right)\;\frac{t^{2}}{∠2}\text{.}$$

in general,

$$\theta \left(x,y,t\right)=\theta_{0}\left(x,t\right)+\theta_{1}\left(x,t\right)+\theta_{2}\left(x,t\right)+\ldots$$

$$\theta \left(x,y,t\right)=-e^{-2\nu t}\;\sin\left(x+y\right)\text{.}$$

similarly, Applying Elzaki transform on Equation (2.59),

$$E\left[\eta_{t}\right]=E\left[\frac{1}{Re}\left\{\eta_{xx}+\eta_{yy}\right\}-\theta \;\eta_{x}-\eta \;\eta_{y}\right]\text{,}$$

$$\Rightarrow \frac{1}{\nu }E\left[\eta \left(x,y,t\right)\right]-\nu \;\eta \left(x,y,0\right)=E\left[\frac{1}{Re}\left\{\eta_{xx}+\eta_{yy}\right\}-\theta \;\eta_{x}-\eta \;\eta_{y}\right]\text{,}$$

$$\Rightarrow E\left[\eta \left(x,y,t\right)\right]=\nu^{2}\eta \left(x,y,0\right)+\nu E\left[\frac{1}{Re}\left\{\eta_{xx}+\eta_{yy}\right\}-\theta \;\eta_{x}-\eta \;\eta_{y}\right]\text{,}$$

$$\Rightarrow \eta \left(x,y,t\right)=\eta \left(x,y,0\right)E^{-1}\left(\nu^{2}\right)+E^{-1}\left[\nu E\left[\frac{1}{Re}\left\{\eta_{xx}+\eta_{yy}\right\}-\theta \;\eta_{x}-\eta \;\eta_{y}\right]\right]\text{,}$$

(2.72) $$\eta \left(x,y,t\right)=\sin\left(x+y\right)+E^{-1}\left[\nu E\left[\frac{1}{Re}\left\{\eta_{xx}+\eta_{yy}\right\}-\theta \;\eta_{x}-\eta \;\eta_{y}\right]\right]\text{.}$$

Applying HPM in Equation (2.72)

(2.73) $$\sum_{n=0}^{\infty }p^{n}\eta_{n}\left(x,y,t\right)=-\sin\left(x+y\right)+p\;E^{-1}\left[\nu E\{\frac{1}{Re}\left\{{\left(\sum_{n=0}^{\infty }\eta_{n}\left(x,t\right)\right)}_{xx}+{\left(\sum_{n=0}^{\infty }\eta_{n}\left(x,t\right)\right)}_{yy}\right\}-I_{n}\left(\theta ,\eta \right)\right]\text{.}$$

where $I_{n}\left(U,\eta \right)$ is the non-linear term.

$$I_{n}\left(\theta ,\eta \right)=\theta \;\eta_{x}+\eta \;\eta_{y}\text{.}$$

Comparing powers of p in Equation (2.73),

$$p^{0}:\eta_{0}\left(x,y,t\right)=\sin\left(x+y\right)\text{.}$$

(2.74) $$p^{1}:\eta_{1}\left(x,y,t\right)=E^{-1}\left[\nu E\left\{\frac{1}{Re}\left\{{\left(\eta_{0}\left(x,t\right)\right)}_{xx}+{\left(\eta_{0}\left(x,t\right)\right)}_{yy}\right\}-I_{0}\left(\theta ,\eta \right)\right\}\right]\text{.}$$

where, $I_{0}\left(\theta ,\eta \right)=\theta_{0}{\left(\eta_{0}\right)}_{x}+\eta_{0}{\left(\eta_{0}\right)}_{y}$ = 0.

From Equation (2.74),

$$\eta_{1}\left(x,y,t\right){=E}^{-1}\left[\nu E\left\{\frac{1}{Re}\left(-2\;\sin\left(x+y\right)\right)\right\}\right]\text{,}$$

$$\Rightarrow \eta_{1}\left(x,y,t\right){=-\frac{2}{Re}\sin\left(x+y\right)E}^{-1}\left[\nu E\left\{1\right\}\right]\text{,}$$

$$\Rightarrow \eta_{1}\left(x,y,t\right){=-\frac{2}{Re}\sin\left(x+y\right)E}^{-1}\left[\nu^{3}\}\right]\text{,}$$

$$\Rightarrow \eta_{1}\left(x,y,t\right)=-\frac{2t}{Re}\sin\left(x+y\right)\text{.}$$

(2.75) $$p^{2}:\eta_{2}\left(x,y,t\right)=E^{-1}\left[\nu E\left\{\frac{1}{Re}\left\{{\left(\eta_{1}\left(x,t\right)\right)}_{xx}+{\left(\eta_{1}\left(x,t\right)\right)}_{yy}\right\}-I_{1}\left(\theta ,\eta \right)\right\}\right]\text{.}$$

where, $I_{1}\left(\theta ,\eta \right)=\left[\theta_{1}{\left(\eta_{0}\right)}_{x}+\theta_{0}{\left(\eta_{1}\right)}_{x}\right]+\left[\eta_{0}{\left(\eta_{0}\right)}_{y}+\eta_{1}{\left(\eta_{0}\right)}_{y}\right]=0\text{.}$.

From Equation (2.75),

$$\eta_{2}\left(x,y,t\right)=E^{-1}\left[\nu E\left\{\frac{4t}{Re^{2}}\sin\left(x+y\right)\right\}\right]\text{,}$$

$$\Rightarrow \eta_{2}\left(x,y,t\right)=\frac{4}{Re^{2}}\sin\left(x+y\right)\;\frac{t^{2}}{∠2}\text{,}$$

in general, $\eta \left(x,y,t\right)=\eta_{0}\left(x,t\right)+\eta_{1}\left(x,t\right)+\eta_{2}\left(x,t\right)+\ldots$.

$$\Rightarrow \eta \left(x,y,t\right)=e^{-2\nu t}\;\sin\left(x+y\right)\text{.}$$

Exact solutions are as follows:

$\theta \left(x,y,t\right)=-e^{-2\nu t}\;\sin\left(x+y\right)$ and $\eta \left(x,y,t\right)=e^{-2\nu t}\;\sin\left(x+y\right)\text{.}$.

### 3.3 Convergence analysis

$\mu_{0}=\frac{\left\|\theta_{1}\right\|}{\left\|\theta_{0}\right\|}$ = 4.0000E-05 < 1, $\mu_{1}=\frac{\left\|\theta_{2}\right\|}{\left\|\theta_{1}\right\|}$ = 2.0000E-05 < 1

$\mu_{2}=\frac{\left\|\theta_{3}\right\|}{\left\|\theta_{2}\right\|}$ = 1.3333E-05 < 1, $\mu_{3}=\frac{\left\|\theta_{4}\right\|}{\left\|\theta_{3}\right\|}$ = 1.0000E-05 < 1

and $\nu_{0}=\frac{\left\|\eta_{1}\right\|}{\left\|\eta_{0}\right\|}$ = 4.0000E-05 < 1, $\nu_{1}=\frac{\left\|\eta_{2}\right\|}{\left\|\eta_{1}\right\|}$ = 2.0000E-05 < 1

$\nu_{2}=\frac{\left\|\eta_{3}\right\|}{\left\|\eta_{2}\right\|}$ = 1.3333E-05 < 1, $\nu_{3}=\frac{\left\|\eta_{4}\right\|}{\left\|\eta_{3}\right\|}$ = 1.0000E-05 < 1

$\mu_{i}^{\prime }s$ and ${\nu_{i}}^{\prime }s$ for i≥0 and $t\leq \left(\frac{j}{2}\right)$ for 0<j<1 are less than one. Hence convergence condition is completed.

## 4 Conclusion

In present paper, a comparison of two methods named Sumudu HPM and Elzaki HPM is provided regarding coupled 1D and 2D Burgers’ equations. In this comparative study, it got confirmed that analytical solutions obtained from SHPM and EHPM are same. These methods are easy to implement and produced same result, which affirms accuracy and efficacy of implemented methods.

The compatibility of approx. and exact solutions for Examples 1 through 3 is demonstrated in Fig. 1 through 8 at various time scales. Aspect of numerical convergence is further asserted for the suggested regime via Table 5, Table 8 Table 5, Table 8 show that as the number of grid points increased, the error decreased, which is evidence of the numerical convergence. Via Tables 5 and it is notified that at t = 1, 2, and 3, error got reduced up to $10^{-16}$. Via Tables 8 and it is notified that at t = 1, error is reduced up to $10^{-12}$, at t = 1.3, error got reduced up to $10^{-11}$, and at t = 1.5, error got reduced up to $10^{-11}$.

The novelty this research is to identify an approx. analytical solution and an exact solution to aforementioned coupled Burgers’ equations. Creating a numerical software to solve fractional differential equations of a complex kind is not an easy undertaking. To deal with such fractional equations, it is therefore necessary to build such Integral transform-based approaches. Furthermore, discretization always introduces a small margin of error in numerical approaches, but in the suggested regime, discretization is not necessary. Because of this, such designed systems do not contain any discretization-related errors.

### 4.1 Future scope of the study

Proposed regimes provide an easy and efficient way to deal with analytical solution of PDEs as developing programming to obtain numerical approximation is not an easy task always. These regimes will surely help researchers to fetch analytical solution of some other complex natured PDEs.

## Author contribution statement

Mamta Kapoor: Conceived and designed the experiments; Performed the experiments; Analyzed and interpreted the data; Contributed reagents, materials, analysis tools or data; Wrote the paper.

Varun Joshi: Contributed reagents, materials, analysis tools or data.

## Data availability statement

No data was used for the research described in the article.

## Declaration of competing interest

The authors declare that they have no known competing financial interests or personal relationships that could have appeared to influence the work reported in this paper.
